# Acceptance of coronavirus disease 2019 (COVID-19) vaccines among healthcare workers: A meta-analysis

**DOI:** 10.3389/fpubh.2022.881903

**Published:** 2022-09-16

**Authors:** Linlin Wang, Ye Wang, Xianbin Cheng, Xingzhao Li, Yanyan Yang, Jun Li

**Affiliations:** ^1^Department of Ultrasound, China-Japan Union Hospital of Jilin University, Changchun, China; ^2^Department of Pediatrics, China-Japan Union Hospital of Jilin University, Changchun, China; ^3^Department of Gastrointestinal Colorectal and Anal Surgery, China-Japan Union Hospital of Jilin University, Changchun, China; ^4^Department of Hematology and Oncology, China-Japan Union Hospital of Jilin University, Changchun, China

**Keywords:** COVID-19, vaccines, meta-analysis, seasonal influenza, healthcare workers

## Abstract

**Background:**

The coronavirus disease 2019 (COVID-19) pandemic has posed increasing challenges to global health systems. Vaccination against COVID-19 can effectively prevent the public, particularly healthcare workers (HCWs), from being infected by this disease.

**Objectives:**

We aim to understand the factors influencing HCWs' acceptance of COVID-19 vaccines.

**Methods:**

We searched PubMed, Embase and Web of Science to collect literature published before May 15, 2022, about HCWs' acceptance of COVID-19 vaccines. The Newcastle–Ottawa quality assessment scale was used to assess the risk of bias and the quality of the included studies. We utilized Stata 14.0 software for this meta-analysis with a random-effects model, and odds ratios (ORs) with 95% confidence intervals (CIs) were reported. This meta-analysis was conducted in alignment with the preferred reporting items for systematic review and meta-analysis (PRISMA) guideline.

**Results:**

Our meta-analysis included 71 articles with 93,508 HCWs involved. The research showed that the acceptance of vaccines had significantly increased among HCWs compared to non-HCWs (OR = 1.91, 95% CI: 1.16–3.12). A willingness to undergo COVID-19 vaccination was observed in 66% (95% CI: 0.61–0.67) of HCWs. Among the HCWs involved, doctors showed a generally increased intention to be vaccinated compared with nurses (OR = 2.22, 95% CI: 1.71–2.89). Additionally, males were found to hold more positive attitudes toward vaccination than females (OR = 1.81, 95% CI: 1.55–2.12). When the effectiveness of COVID-19 vaccines was improved, the vaccination acceptance of HCWs was greatly increased accordingly (OR = 5.03, 95% CI: 2.77–9.11). The HCWs who were willing to vaccinate against seasonal influenza showed an increased acceptance of COVID-19 vaccines (OR = 3.52, 95% CI: 2.34–5.28). Our study also showed that HCWs who were willing to be vaccinated against COVID-19 experienced a reduced rate of severe acute respiratory syndrome coronavirus 2 (SARS-CoV-2) infection (OR = 0.78, 95% CI: 0.66–0.92).

**Conclusions:**

Our analysis revealed that the five factors of occupation, gender, vaccine effectiveness, seasonal influenza vaccines, and SARS-CoV-2 infection presumably affected the acceptance of COVID-19 vaccines among HCWs. It is essential to boost the confidence of HCWs in COVID-19 vaccines for the containment of the epidemic.

## Introduction

### Rationale

On March 16, 2020, the first mRNA vaccine for coronavirus disease 2019 (COVID-19) developed by Moderna entered the clinical trial stage in the United States. Subsequently, various COVID-19 vaccines, including DNA-based vaccines, have been popularized throughout the world (1). Developing safe and effective vaccines to promote large-scale vaccination is probably the most effective way for humankind to fight against COVID-19 (2).

In 2022, millions of doses of COVID-19 vaccines are now administered each day globally (3). Surprisingly, numerous people showed distrust and concerns about COVID-19 vaccines (4). A large number of studies have shown that some healthcare workers (HCWs) remain skeptical about whether to receive COVID-19 vaccination (5). In one survey, approximately one-sixth of HCWs claimed that they would not choose to be vaccinated against COVID-19 even if mandated (6). The risk of the members of HCWs infected with COVID-19 was nearly three times that of the non-HCWs (7). In some countries, approximately 10% of HCWs are infected with SARS-CoV-2 (8). The acceptance of COVID-19 vaccines among non-HCWs can be easily affected by HCWs; in particular, HCWs with a negative attitude tend not to recommend vaccines to patients (9).

### Objectives

We aim, through meta-analysis, to understand the factors influencing HCWs' acceptance of vaccination against COVID-19. Our study may provide insights for promoting future immunization programs worldwide.

## Materials and methods

### Eligibility criteria

Studies meeting the following criteria were included in the meta-analysis: (1) the content must include the acceptance of HCWs about COVID-19 vaccines, (2) the number of HCWs who are willing and unwilling (including refusal and hesitation) to vaccinate should be recorded separately, and (3) the sample sizes of both the experimental group and the control group were more than 10.

Information from abstracts, comments, reviews, posters and case reports was excluded.

### Information sources

All the literature published before May 15, 2022, about the acceptance of HCWs toward COVID-19 vaccines was searched in PubMed, Embase, and Web of Science, regardless of the language of the literature, to collect the most useful information.

### Search strategy

The method of “key words” + “free words” was adopted for retrieval. Search terms were limited to the titles and abstracts. Detailed strategies are listed in Supplementary File 1.

### Study selection process

Literature collected from the database was imported into NoteExpress software for filtration. After deleting duplicated literature, we first read the titles and abstracts before we eliminated irrelevant pieces. Articles that did not meet the requirements were then further screened based on the abstracts or the full text. Articles that were fairly related were adopted for subsequent data selection.

### Data selection process and items

Data extraction was completed independently by two authors. When those two authors disagreed on data selection, they would debate the problem before delivering it to a third author for the final conclusion.

The following data were recorded: the number of HCWs willing and unwilling to be vaccinated against COVID-19; the number of HCWs who had been vaccinated against seasonal influenza in 2019–2020 and who preferred to be vaccinated against the same disease in 2020–2021; the number of HCWs in favor of compulsory COVID-19 vaccination; the number of doctors and nurses willing to receive COVID-19 vaccines; the number of non-HCWs willing to be vaccinated with COVID-19; the number of HCWs willing to be vaccinated with different effective rates (bounded by 70%); the gender, age, and education level of HCWs; the number of HCWs afflicted with chronic diseases; the number of HCWs who contacted closely with COVID-19 patients; and the number of people vaccinated against influenza and the number of COVID-19 cases in the two groups of HCWs who were willing and unwilling to be vaccinated against COVID-19. If an article could extract several groups of data without intersection or the data record research results under different conditions, they were represented by “-A,” “-B” or “-C.”

### Study risk of bias assessment

The quality and the risk of bias of the included studies were independently assessed using the Newcastle–Ottawa quality assessment scale. A low risk of bias and high quality were considered if the overall score was equal to or above seven. The assessment was completed by one author and reviewed by another.

### Reporting bias assessment

Egger's test was used for quantitative analysis. A *p*-value < 0.05 indicates the presence of bias.

### Synthesis methods

The *I*^2^ statistic was used to quantify the heterogeneity among studies. An *I*^2^ value <50% indicated mild heterogeneity, while an *I*^2^ value ≥ 75% suggested significant heterogeneity. Moderate heterogeneity was considered if 50% ≤ *I*^2^ <75%. We conducted subgroup analysis to explore the source of heterogeneity. A random-effects model was used to estimate the effect value. Stata 14.0 software was applied for all analyses. A *p*-value of z test < 0.05 was considered to be statistically significant.

### Effect measures and certainty assessment

In this study, the ratio and odds ratio (OR) were used for data analysis, and the confidence interval (CI) was 95%.

## Results

### Study selection

**Figure 1:**
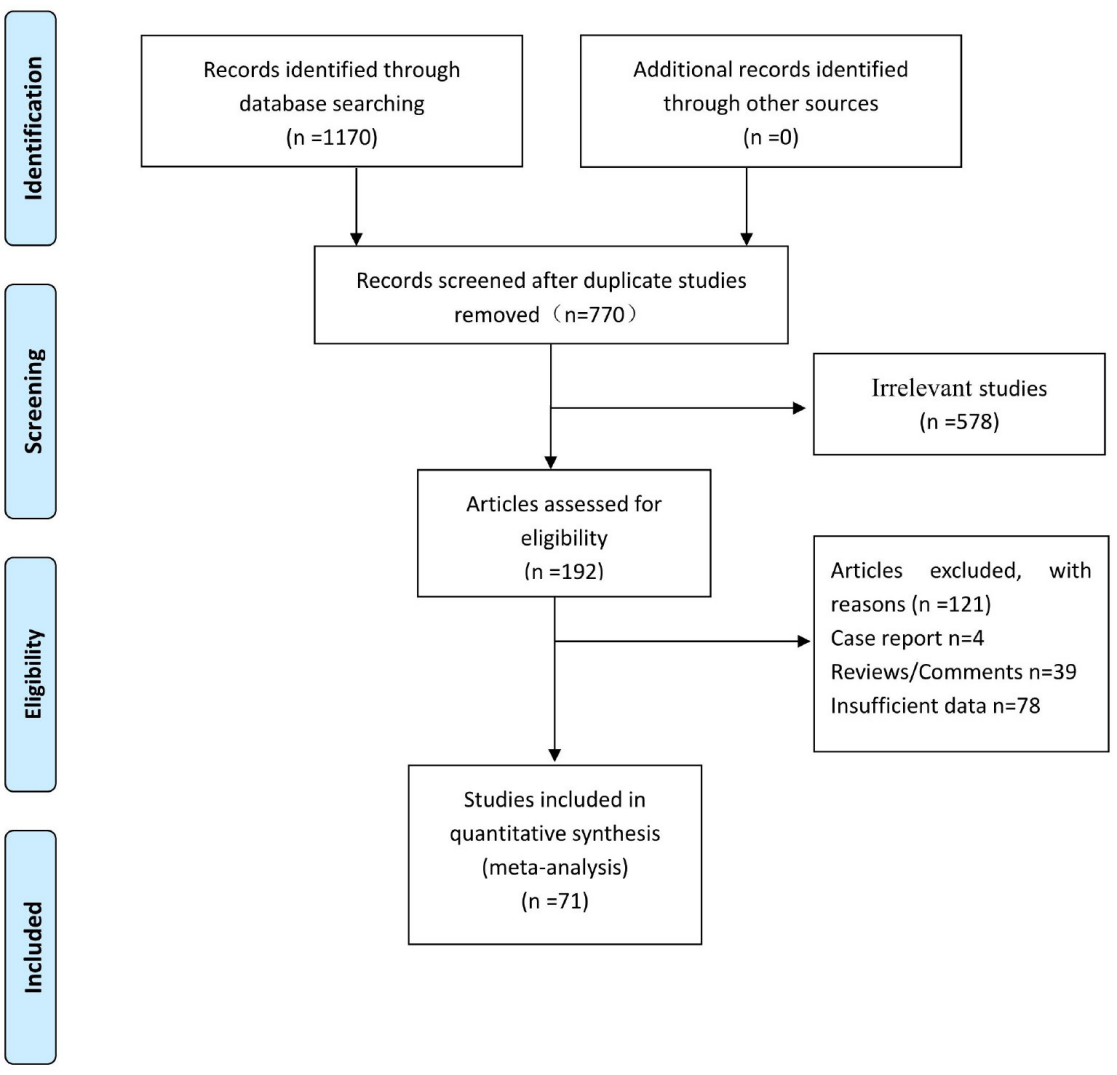
Flow diagram of study selection.

A total of 1,170 studies were searched in the database, of which 400 duplicated studies were deleted with NoteExpress software. According to the titles and abstracts, 578 articles irrelevant to this study were eliminated. Of the remaining 192 papers, 121 were excluded after further screening, including comments, reviews, case reports, and papers with insufficient data. Seventy-one articles were finalized for inclusion in our meta-analysis. The flow diagram of the study selection is shown in Figure 1.

### Study characteristics

The HCWs in our study came from various occupations, including doctors, nurses, paramedics, medical teachers, and students. The whole sample we extracted from the literature included 75,345 HCWs and 13,513 non-HCWs, covering 40 countries and regions.

### Risk of bias in studies

All the studies included in the Newcastle–Ottawa quality assessment scale indicated a fairly low risk of bias and high quality (Supplementary Table 1).

### Results of individual studies

The results of individual studies are presented in structured tables. The information of HCWs and non-HCWs is listed in Table 1. Among HCWS, information on people's willingness to receive COVID-19 vaccines is shown in Table 2.

**Table 1:** The characteristics of HCWs and non-HCWs.

**Reference**	**Rigion**	**Publication year**	**Study period**	**HCWs**	**The number of HCWs in favor of compulsory vaccination**	**Doctors**	**Nurses**	**Non-HCWs**	**Vaccine effectiveness (over 70%)**	**Willing to receive COVID-19 vaccines among HCWs**	**Willing to receive COVID-19 vaccines among doctors**	**Willing to receive COVID-19 vaccines among nurses**	**Willing to receive COVID-19 vaccines among non-HCWs**	**Vaccination against seasonal influenza in 2019–2020 among HCWs**	**Willing to receive seasonal influenza vaccines in 2020–2021 among HCWs**
Mascarenhas et al. (6)	America	2021	NA	245	98	NA	NA	NA	NA	136	NA	NA	NA	148	178
Qattan et al. (10)	Saudi Arabia	2021	2020.12.8–2020.12.14	673	NA	NA	NA	NA	NA	340	NA	NA	NA	NA	NA
Papagiannis et al. (11)	Greece	2021	2020.12.15–2020.12.22	340	NA	NA	NA	NA	NA	267	NA	NA	NA	NA	251
Nzaji et al. (12)	Congo	2020	2020.3.20–2020.4.30	613	NA	NA	NA	NA	NA	170	NA	NA	NA	NA	NA
Harapan et al. (13)-A	Indonesia	2020	2020.3.25–2020.4.6	264	NA	NA	NA	1,095	Yes	252	NA	NA	1,016	NA	NA
Harapan et al. (13)-B	Indonesia	2020	2020.3.25–2020.4.6	264	NA	NA	NA	1,095	No	193	NA	NA	718	NA	NA
Singhania et al. (14)	India	2021	2021.1.20–2021.1.24	721	NA	615	56	NA	NA	572	496	32	NA	NA	NA
Kanyike et al. (15)	Uganda	2021	2021.3.15–2021.3.21	600	NA	NA	NA	NA	NA	224	NA	NA	NA	NA	NA
Chew et al. (16)	Asia-Pacific	2021	2020.12.12–2020.12.21	1,720	NA	892	404	NA	NA	1,655	859	389	NA	NA	NA
Papagiannis et al. (17)	Greece	2020	2020.2.10–2020.2.25	461	NA	140	215	NA	NA	200	85	73	NA	NA	NA
Shaw et al. (18)	America	2021	2020.11.23–2020.12.5	5,287	NA	NA	NA	NA	NA	3,032	NA	NA	NA	NA	NA
Szmyd et al. (19)	Poland	2021	2020.12.22–2021.1.8	387	NA	NA	NA	1,913	NA	321	NA	NA	1,039	NA	NA
Ledda et al. (20)	Italy	2021	2020.9.1–2020.12.20	787	NA	324	357	NA	NA	593	261	251	NA	NA	NA
Verger et al. (21)-A	France	2021	2020.10.1–2020.11.30	1,209	NA	NA	NA	NA	NA	910	NA	NA	NA	1,031	NA
Verger et al. (21)-B	Belgium	2021	2020.10.1–2020.11.30	414	NA	NA	NA	NA	NA	315	NA	NA	NA	347	NA
Verger et al. (21)-C	Canada	2021	2020.10.1–2020.11.30	1,055	NA	NA	NA	NA	NA	743	NA	NA	NA	636	NA
Gennaro et al. (22)	Italy	2021	2020.10.1–2021.11.1	1,723	NA	NA	NA	NA	NA	1,115	NA	NA	NA	810	1,364
Bauernfeind et al. (23)	Germany	2021	2020.12.12–2020.12.21	2,454	NA	423	629	NA	NA	1,469	350	335	NA	1,025	1,325
Abuown et al. (24)	England	2021	2020.12.1–2020.12.21	514	NA	NA	NA	NA	NA	304	NA	NA	NA	NA	NA
Fares et al. (25)	Egypt	2021	2020.12.1–2021.1.31	385	NA	205	89	NA	NA	80	49	10	NA	NA	NA
Manning et al. (26)	America	2021	2020.8.10–2020.9.14	1,212	NA	NA	NA	NA	NA	561	NA	NA	NA	NA	NA
Shekhar et al. (27)	America	2021	2020.10.7–2020.11.9	3,479	NA	NA	NA	NA	NA	1,247	NA	NA	NA	3,363	NA
Dzieciolowska et al. (28)	Canada	2021	2020.12.15–2020.12.28	2,761	NA	NA	NA	NA	NA	2,233	NA	NA	NA	NA	NA
Theodore et al. (29)	America	2020	2020.4.26–2020.7.22	121	NA	NA	NA	NA	NA	94	NA	NA	NA	NA	NA
Maraqa et al. (30)	Palestine	2021	2020.12.25–2021.1.6	1,159	NA	374	483	NA	NA	438	231	118	NA	NA	NA
Lucia et al. (31)	America	2020	NA	167	110	NA	NA	NA	NA	126	NA	NA	NA	NA	NA
Gadoth et al. (32)	America	2021	2020.9.24–2020.10.16	540	NA	201	207	NA	NA	447	187	147	NA	NA	NA
Maltezou et al. (33)	Greece	2021	2020.9.1–2020.10.31	1,571	1,299	480	607	NA	NA	803	343	261	NA	NA	NA
Janssens et al. (34)	Germany	2021	2020.12.1–2020.12.31	2,305	NA	NA	NA	NA	NA	1,471	NA	NA	NA	NA	NA
Ahmed et al. (35)	Saudi Arabia	2021	2020.10.1–2020.10.31	236	NA	38	146	NA	NA	115	18	69	NA	NA	NA
Kwok et al. (36)	Hong Kong	2021	2020.3.15–2020.4.30	1,205	NA	NA	NA	NA	NA	759	NA	NA	NA	590	NA
Wang et al. (37)	Hong Kong	2020	2020.2.26–2020.3.31	806	NA	NA	NA	NA	NA	322	NA	NA	NA	383	360
Konopinska et al. (38)	Poland	2021	2021.1.1–2021.1.31	126	NA	NA	NA	NA	NA	90	NA	NA	NA	NA	NA
Elhadi et al. (39)-A	Libya	2021	2020.12.1–2020.12.18	3,967	NA	1,394	821	NA	Yes	3,174	1,138	643	NA	NA	NA
Elhadi et al. (39)-B	Libya	2021	2020.12.1–2020.12.18	3,967	NA	1,394	821	NA	No	1,552	494	314	NA	NA	NA
Szmyd et al. (40)	Poland	2021	2020.12.22–2020.12.25	687	NA	NA	NA	1,284	NA	632	NA	NA	763	NA	NA
Gonullu et al. (41)	Turkey	2021	2020.11.1–2020.11.15	506	303	NA	NA	NA	NA	420	NA	NA	NA	198	354
Socarras et al. (42)-A	Columbia	2021	2021.1.1–2021.1.31	1,066	NA	NA	NA	NA	Yes	821	NA	NA	NA	NA	NA
Socarras et al. (42)-B	Columbia	2021	2021.1.1–2021.1.31	1,066	NA	NA	NA	NA	No	967	NA	NA	NA	NA	NA
Kuter et al. (43)	America	2021	2020.11.13–2020.12.6	12,034	NA	NA	NA	NA	NA	7,284	NA	NA	NA	NA	NA
Yu et al. (44)	China	2021	2020.10.1–2020.11.30	2,264	NA	362	1,902	NA	NA	294	55	239	NA	NA	NA
Hoke et al. (45)	America	2021	2020.5.1–2020.5.31	350	NA	NA	NA	NA	NA	297	NA	NA	NA	NA	NA
Giuseppe et al. (46)	Italy	2021	2020.9.14–2020.11.30	779	NA	437	194	NA	NA	629	395	132	NA	NA	NA
Kaplan et al. (47)	Turkey	2021	2020.12.25–2020.12.31	1,574	NA	1,115	275	NA	NA	1,331	1,003	183	NA	NA	NA
Kose et al. (48)	Turkey	2020	2020.9.17–2020.9.20	1,138	NA	53	306	NA	NA	781	27	200	NA	312	NA
Saied et al. (49)	Egypt	2021	2021.1.1–2021.1.31	2,133	1,487	NA	NA	NA	NA	746	NA	NA	NA	112	51
Dror et al. (50)	Israel	2020	2020.3.19–2020.3.25	549	NA	338	211	1,112	NA	393	264	129	834	NA	NA
Unroe et al. (51)	America	2021	2020.11.14–2020.11.17	8,243	NA	NA	NA	NA	NA	5,705	NA	NA	NA	NA	NA
Kukreti et al. (52)	Taiwan	2021	2020.9.24–2020.12.31	500	NA	NA	NA	238	NA	117	NA	NA	73	NA	NA
Gakuba et al. (53)	France	2021	2021.2.1–2021.2.28	61	NA	NA	NA	NA	NA	34	NA	NA	NA	NA	NA
Wang et al. (54)	China	2021	2020.9.15–2020.9.20	3,634	NA	1,123	1,841	NA	NA	2,874	929	1,400	NA	NA	NA
Yurttas et al. (55)	Turkey	2021	2021.1.4–2021.1.13	320	113	NA	NA	732	NA	168	NA	NA	214	NA	NA
Noushad et al. (56)	Twelve countries	2022	2021.2–2021.4	2,962	NA	NA	NA	NA	NA	2,038	NA	NA	NA	NA	NA
Dkhar et al. (57)	India	2022	NA	511	NA	NA	NA	NA	NA	340	NA	NA	NA	NA	NA
Adeniyi et al. (58)	South Africa	2021	2020.11–2020.12	1,308	NA	176	591	NA	NA	1,179	158	527	NA	NA	NA
Ayele et al. (59)	Ethiopia	2021	2021.3.1–2021.3.30	422	NA	60	148	NA	NA	191	39	52	NA	NA	NA
Vignier et al. (60)	French Guiana	2021	2021.1.22–2021.3.26	579	NA	NA	NA	NA	NA	373	NA	NA	NA	183	140
Do et al. (61)	America	2021	2020.12.10–2020.12.20	1,076	NA	63	275	NA	NA	563	52	144	NA	NA	NA
Khan et al. (62)	Pakistan	2022	NA	248	NA	NA	NA	NA	NA	219	NA	NA	NA	NA	NA
Wiysonge et al. (63)	South Africa	2022	2021.3–2021.5	395	NA	49	191	NA	NA	233	44	97	NA	NA	NA
Koh et al. (64)	Singapore	2022	2021.5–2021.6	528	NA	NA	NA	NA	NA	501	NA	NA	NA	NA	487
Sharaf et al. (65)	Egypt	2022	2021.8–2021.10	171	NA	NA	NA	NA	NA	78	NA	NA	NA	NA	NA
Raja et al. (66)	Sudan	2022	2021.6.30–2021.7.11	217	NA	NA	NA	NA	NA	121	NA	NA	NA	NA	NA
Pal et al. (67)	America	2021	2021.2.1–2021.3.31	1,358	NA	NA	NA	NA	NA	1,251	NA	NA	NA	NA	NA
Saddik et al. (68)	United Arab Emirates	2021	2020.11.20–2021.1.3	517	NA	NA	NA	NA	NA	312	NA	NA	NA	NA	NA
Hara et al. (69)	Japan	2021	2021.1.19	1,030	NA	120	369	6,180	NA	477	65	168	3,003	NA	NA
Boche et al. (70)	Ethiopia	2022	2021.6.30–2021.7.30	319	NA	NA	NA	NA	NA	232	NA	NA	NA	NA	NA
Thomas et al. (71)	America	2022	2021.3.12–2021.4.22	505	NA	NA	NA	NA	NA	457	NA	NA	NA	NA	NA
Otiti-Sengeri et al. (72)	Uganda	2022	2021.6–2021.8	300	NA	NA	NA	NA	NA	293	NA	NA	NA	NA	NA
Rosental et al. (73)	Israel	2021	2020.8.27–2020.9.28	628	NA	321	307	NA	NA	517	283	234	NA	NA	NA
Kashif et al. (74)	Pakistan	2021	2020.12.19–2021.1.10	208	NA	NA	NA	196	NA	112	NA	NA	56	NA	NA
Kateeb et al. (75)	Palestine	2021	2021.2–2021.3	417	NA	NA	NA	NA	NA	241	NA	NA	NA	NA	NA
Xu et al. (76)	China	2021	2021.4.16–2021.4.18	1,051	NA	NA	NA	NA	NA	906	NA	NA	NA	NA	NA
Yilma et al. (77)	Ethiopia	2022	2021.2–2021.4	1,314	NA	NA	NA	NA	NA	982	NA	NA	NA	NA	NA
Li et al. (78)	China	2021	2021.1.20–2021.2.20	1,779	NA	300	1,317	NA	NA	1,670	290	1,232	NA	NA	NA
Yurttas et al. (55)	Turkey	2021	2021.1.4–2021.1.13	320	NA	NA	NA	763	NA	168	NA	NA	264	NA	NA

HCWs, Healthcare workers; NA, not applicable; -A, -B or –C, an article could extract several groups of data without intersection, or the data record research results under different conditions; COVID-19, coronavirus disease 2019.

**Table 2:** The characteristics of HCWs who are willing and unwilling to receive coronavirus disease 2019 vaccines.

**Reference**	**Rigion**	**Publication year**	**Study period**	**HCWs**		**Age**<**40**		**Age**<**50**		**Male**		**Less than bachelor's degree**		**Close contact with COVID-19 patients**		**Chronic diseases**		**Married**		**Willing to receive seasonal influenza vaccines in 2020–2021**		**Vaccination against seasonal influenza in 2019–2020**		**SARS-CoV-2infection**	
				**Willing**	**No**	**Willing**	**No**	**Willing**	**No**	**Willing**	**No**	**Willing**	**No**	**Willing**	**No**	**Willing**	**No**	**Willing**	**No**	**Willing**	**No**	**Willing**	**No**	**Willing**	**No**
Mascarenhas et al. (6)	America	2021	NA	136	109	NA	NA	NA	NA	NA	NA	NA	NA	NA	NA	NA	NA	NA	NA	120	58	100	49	7	18
Qattan et al. (10)	Saudi Arabia	2021	2020.12.8–2020.12.14	340	333	227	225	306	287	228	177	NA	NA	183	144	70	61	234	236	NA	NA	NA	NA	NA	NA
Papagiannis et al. (11)	Greece	2021	2020.12.15–2020.12.22	267	73	NA	NA	NA	NA	142	31	NA	NA	NA	NA	NA	NA	NA	NA	205	43	NA	NA	NA	NA
Nzaji et al. (12)	Congo	2020	2020.3.20–2020.4.30	170	443	118	303	NA	NA	110	202	NA	NA	NA	NA	NA	NA	120	288	NA	NA	NA	NA	NA	NA
Singhania et al. (14)	India	2021	2021.1.20–2021.1.24	572	149	NA	NA	NA	NA	NA	NA	NA	NA	389	112	NA	NA	NA	NA	NA	NA	NA	NA	109	40
Kanyike et al. (15)	Uganda	2021	2021.3.15–2021.3.21	224	376	NA	NA	NA	NA	160	217	NA	NA	NA	NA	NA	NA	20	54	NA	NA	NA	NA	NA	NA
Chew et al. (16)	Asia-Pacific	2021	2020.12.12–2020.12.21	1,655	65	NA	NA	NA	NA	646	24	91	0	NA	NA	561	44	1,019	35	NA	NA	NA	NA	NA	NA
Papagiannis et al. (17)	Greece	2020	2020.2.10–2020.2.25	200	261	NA	NA	NA	NA	69	49	NA	NA	NA	NA	NA	NA	NA	NA	NA	NA	NA	NA	NA	NA
Shaw et al. (18)	America	2021	2020.11.23–2020.12.5	3,032	2,255	NA	NA	NA	NA	992	376	NA	NA	1,670	1,423	NA	NA	NA	NA	NA	NA	NA	NA	NA	NA
Ledda et al. (20)	Italy	2021	2020.9.1–2020.12.20	593	194	259	70	423	164	312	56	NA	NA	NA	NA	230	37	NA	NA	NA	NA	NA	NA	NA	NA
Gennaro et al. (22)	Italy	2021	2020.10.1–2021.11.1	1,115	608	900	389	993	496	538	265	NA	NA	NA	NA	NA	NA	NA	NA	NA	NA	NA	NA	54	33
Bauernfeind et al. (23)	Germany	2021	2020.12.12–2020.12.21	1,469	985	NA	NA	NA	NA	595	188	823	762	777	823	NA	NA	NA	NA	1,004	321	787	238	NA	NA
Fares et al. (25)	Egypt	2021	2020.12.1–2021.1.31	80	305	NA	NA	NA	NA	28	44	3	11	47	111	NA	NA	NA	NA	NA	NA	NA	NA	32	113
Manning et al. (26)	America	2021	2020.8.10–2020.9.14	561	651	455	538	499	600	79	52	NA	NA	NA	NA	NA	NA	NA	NA	NA	NA	NA	NA	NA	NA
Shekhar et al. (27)	America	2021	2020.10.7–2020.11.9	1,247	2,232	640	1,237	867	1,696	425	439	86	241	814	1,402	733	1306	NA	NA	NA	NA	1,237	2,126	31	59
Maraqa et al. (30)	Palestine	2021	2020.12.25–2021.1.6	438	721	NA	NA	382	619	NA	NA	NA	NA	NA	NA	NA	NA	NA	NA	NA	NA	NA	NA	90	172
Lucia et al. (31)	America	2020	NA	126	41	NA	NA	NA	NA	NA	NA	NA	NA	NA	NA	NA	NA	NA	NA	NA	NA	NA	NA	4	1
Maltezou et al. (33)	Greece	2021	2020.9.1–2020.10.31	803	768	334	311	556	539	365	185	NA	NA	456	376	586	374	NA	NA	NA	NA	NA	NA	NA	NA
Ahmed et al. (35)	Saudi Arabia	2021	2020.10.1–2020.10.31	115	121	NA	NA	NA	NA	NA	NA	NA	NA	NA	NA	22	10	NA	NA	NA	NA	NA	NA	NA	NA
Wang et al. (37)	Hong Kong	2020	2020.2.26–2020.3.31	322	484	189	236	267	376	67	39	NA	NA	190	247	83	97	NA	NA	NA	NA	202	181	NA	NA
Gonullu et al. (41)	Turkey	2021	2020.11.1–2020.11.15	420	86	NA	NA	NA	NA	184	25	NA	NA	352	72	75	14	NA	NA	316	38	180	18	57	14
Socarras et al. (42)-A	Columbia	2021	2021.1.1–2021.1.31	821	245	NA	NA	NA	NA	440	123	NA	NA	NA	NA	NA	NA	NA	NA	NA	NA	NA	NA	NA	NA
Socarras et al. (42)-B	Columbia	2021	2021.1.1–2021.1.31	967	99	NA	NA	NA	NA	519	44	NA	NA	NA	NA	NA	NA	NA	NA	NA	NA	NA	NA	NA	NA
Kuter et al. (43)	America	2021	2020.11.13–2020.12.6	7,284	4,750	3,835	2,296	NA	NA	2,064	461	618	893	NA	NA	NA	NA	NA	NA	NA	NA	NA	NA	NA	NA
Giuseppe et al. (46)	Italy	2021	2020.9.14–2020.11.30	629	150	NA	NA	474	104	NA	NA	NA	NA	319	65	127	37	280	73	NA	NA	NA	NA	NA	NA
Kaplan et al. (47)	Turkey	2021	2020.12.25–2020.12.31	1,331	243	612	176	977	224	563	85	NA	NA	768	153	421	51	972	152	NA	NA	NA	NA	214	85
Kose et al. (48)	Turkey	2020	2020.9.17–2020.9.20	781	357	NA	NA	NA	NA	234	79	NA	NA	NA	NA	101	55	NA	NA	NA	NA	NA	NA	NA	NA
Saied et al. (49)	Egypt	2021	2021.1.1–2021.1.31	746	1,387	NA	NA	NA	NA	276	466	NA	NA	NA	NA	NA	NA	NA	NA	23	28	50	62	147	304
Gakuba et al. (53)	France	2021	2021.2.1–2021.2.28	34	27	NA	NA	NA	NA	6	3	NA	NA	NA	NA	NA	NA	NA	NA	NA	NA	NA	NA	NA	NA
Wang et al. (54)	China	2021	2020.9.15–2020.9.20	2,874	760	NA	NA	2,499	703	689	131	422	63	526	136	NA	NA	NA	NA	NA	NA	NA	NA	NA	NA
Noushad et al. (56)	Twelve countries	2022	2021.2–2021.4	2,038	924	NA	NA	1,903	890	853	332	NA	NA	NA	NA	263	116	NA	NA	NA	NA	NA	NA	334	197
Dkhar et al. (57)	India	2022	NA	340	171	NA	NA	NA	NA	132	64	NA	NA	139	84	NA	NA	206	104	NA	NA	NA	NA	73	36
Adeniyi et al. (58)	South Africa	2021	2020.11–2020.12	1,179	129	NA	NA	NA	NA	223	19	352	22	906	103	767	91	NA	NA	NA	NA	NA	NA	356	45
Ayele et al. (59)	Ethiopia	2021	2021.3.1–2021.3.30	191	231	146	202	NA	NA	NA	NA	NA	NA	NA	NA	53	39	112	140	NA	NA	NA	NA	15	24
Vignier et al. (60)	French Guiana	2021	2021.1.22–2021.3.26	373	206	NA	NA	220	165	150	36	NA	NA	NA	NA	NA	NA	NA	NA	127	13	164	19	72	38
Do et al. (61)	America	2021	2020.12.10–2020.12.20	563	513	NA	NA	NA	NA	NA	NA	NA	NA	NA	NA	NA	NA	NA	NA	NA	NA	NA	NA	38	64
Khan et al. (62)	Pakistan	2022	NA	219	29	NA	NA	NA	NA	147	12	NA	NA	NA	NA	NA	NA	NA	NA	NA	NA	NA	NA	102	9
Wiysonge et al. (63)	South Africa	2022	2021.3–2021.5	233	162	NA	NA	NA	NA	NA	NA	100	95	NA	NA	NA	NA	NA	NA	NA	NA	NA	NA	NA	NA
Koh et al. (64)	Singapore	2022	2021.5–2021.6	501	27	NA	NA	NA	NA	64	1	NA	NA	406	18	NA	NA	NA	NA	462	25	NA	NA	NA	NA
Sharaf et al. (65)	Egypt	2022	2021.8–2021.10	78	93	59	73	73	89	19	7	NA	NA	59	71	9	12	NA	NA	NA	NA	NA	NA	39	46
Raja et al. (66)	Sudan	2022	2021.6.30–2021.7.11	121	96	NA	NA	NA	NA	57	43	NA	NA	NA	NA	NA	NA	NA	NA	NA	NA	NA	NA	NA	NA
Pal et al. (67)	America	2021	2021.2.1–2021.3.31	1,251	107	NA	NA	NA	NA	258	15	503	64	691	56	NA	NA	NA	NA	NA	NA	NA	NA	NA	NA
Thomas et al. (71)	America	2022	2021.3.12–2021.4.22	457	48	126	18	NA	NA	70	5	NA	NA	336	33	NA	NA	NA	NA	NA	NA	NA	NA	NA	NA
Xu et al. (76)	China	2021	2021.4.16–2021.4.18	906	145	NA	NA	NA	NA	95	16	69	10	NA	NA	NA	NA	NA	NA	NA	NA	NA	NA	NA	NA
Li et al. (78)	China	2021	2021.1.20–2021.2.20	1,670	109	1,388	88	1,621	107	202	8	255	14	NA	NA	NA	NA	976	80	NA	NA	NA	NA	NA	NA

HCWs, Healthcare workers; NA, not applicable; -A or –B, an article could extract several groups of data without intersection, or the data record research results under different condition; COVID-19, coronavirus disease 2019.

### Reporting biases

We used Egger's test for reporting bias analysis (Supplementary File 2). The study of the acceptance of HCWs with different education levels about COVID-19 vaccines showed a slight bias (*p* = 0.049), while other results carried no significant bias.

### Certainty of evidence and results of syntheses

We considered the continent where the study was conducted as the basis of subgroup division and explored the source of heterogeneity through subgroup analysis (Figures 2–10). We found that the heterogeneity in some subgroups remained high.

**Figure 2:**
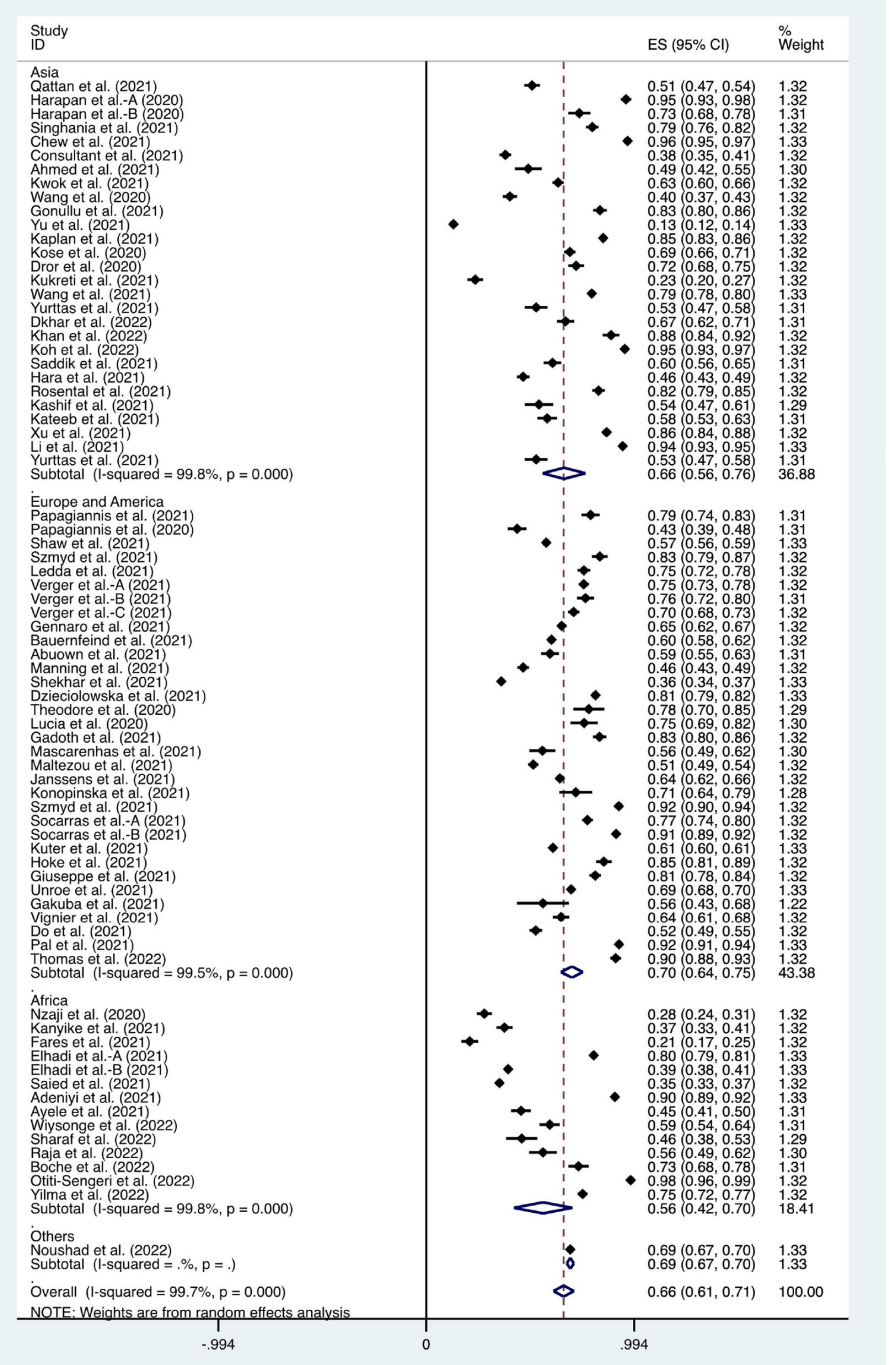
Forest plot of the acceptance of coronavirus disease 2019 vaccines by healthcare workers.

**Figure 3:**
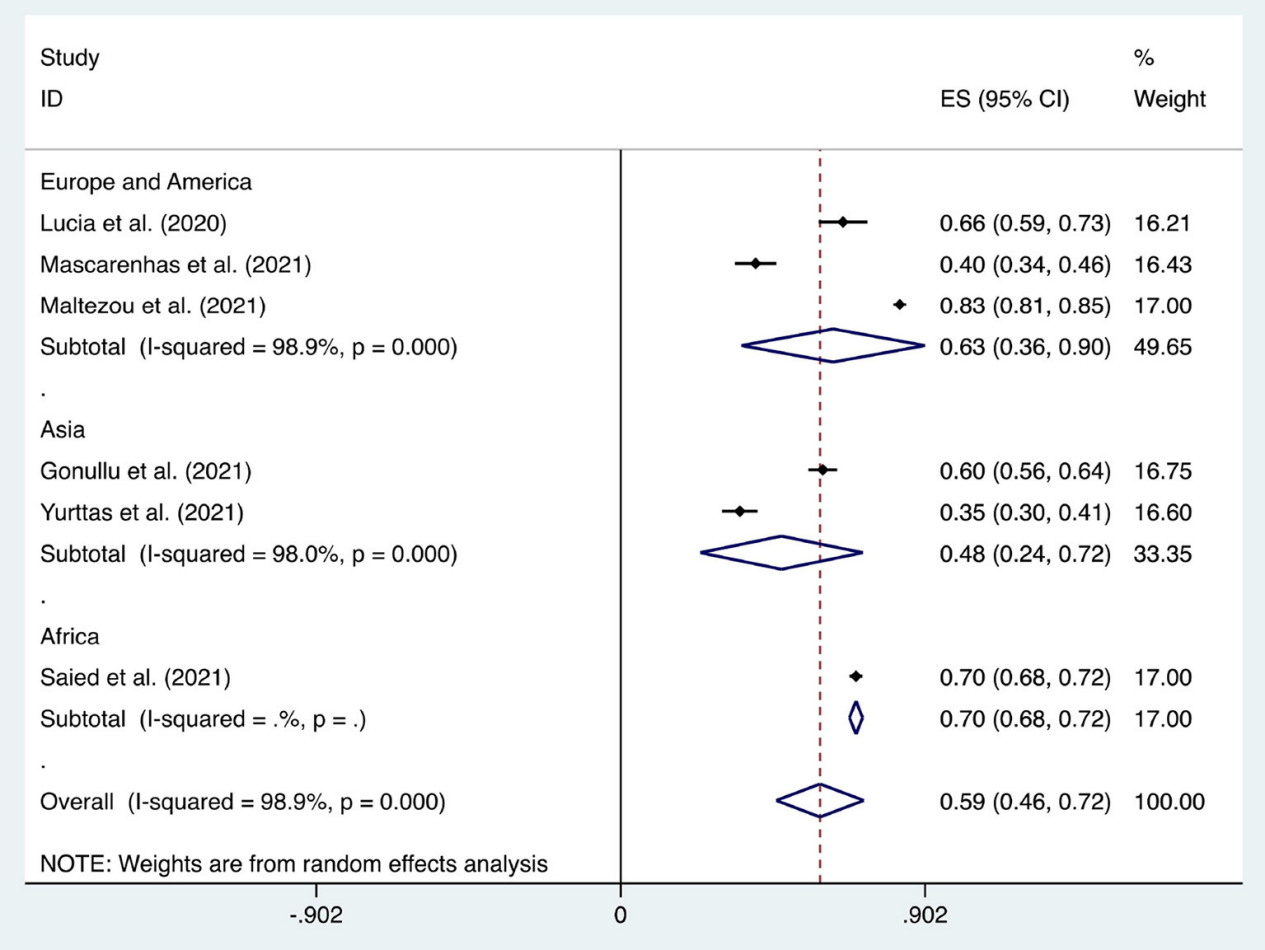
Forest plot of the acceptance of healthcare workers of compulsory vaccination.

**Figure 4:**
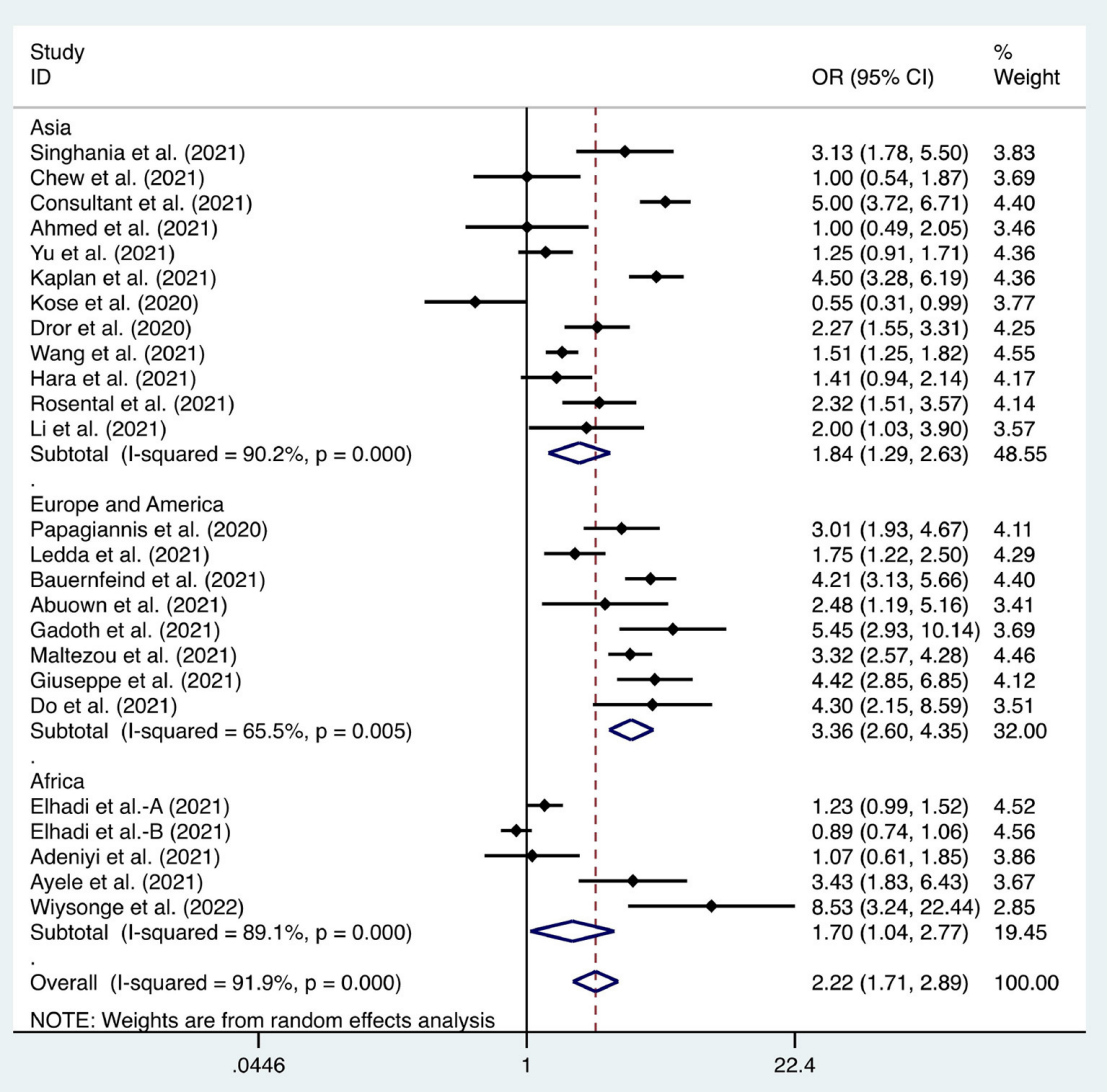
Forest plot of the difference in the willingness between doctors and nurses to receive coronavirus disease 2019 vaccines.

**Figure 5:**
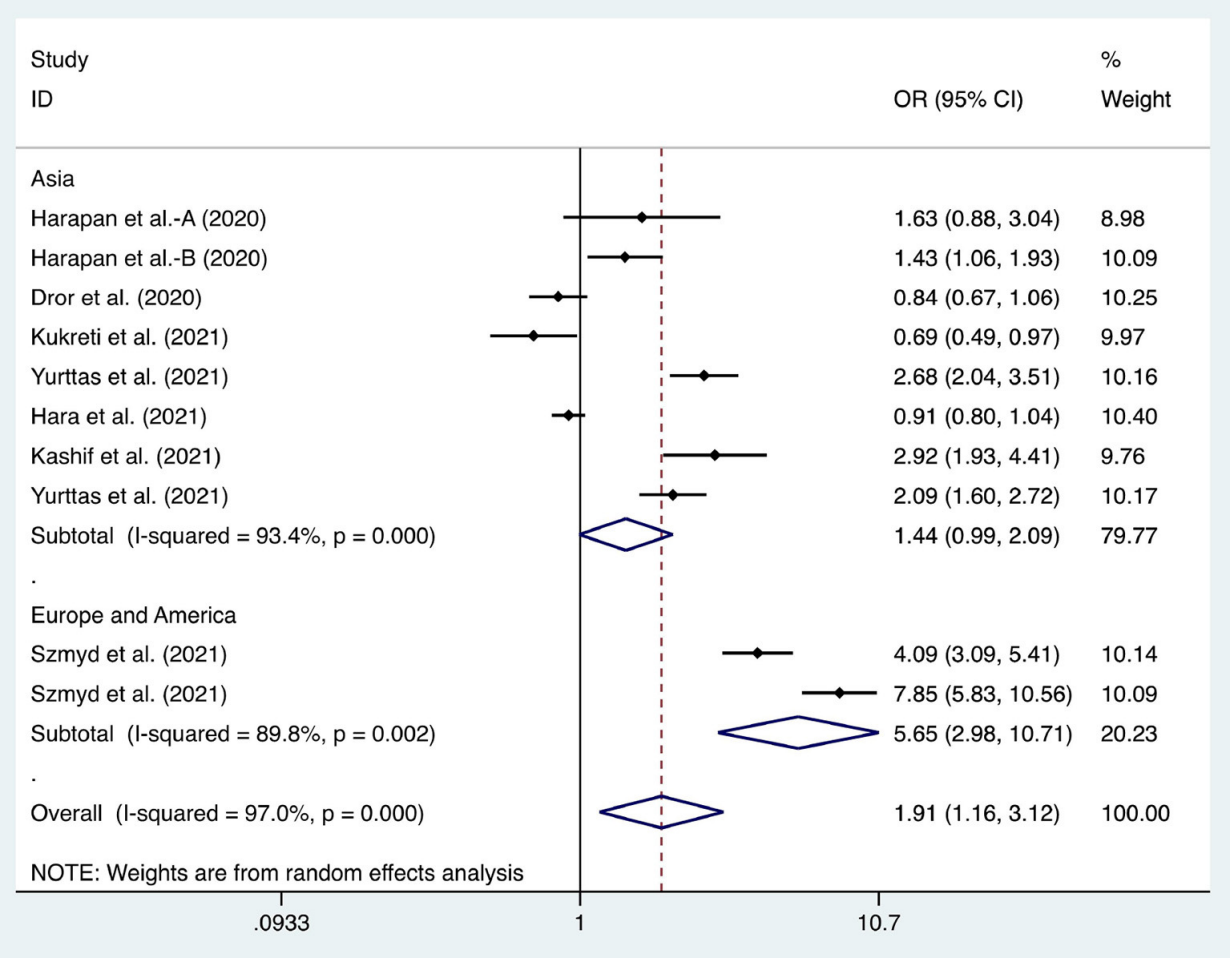
Forest plot of the willingness of healthcare workers (HCWs) and non-HCWs to receive coronavirus disease 2019 vaccines.

**Figure 6:**
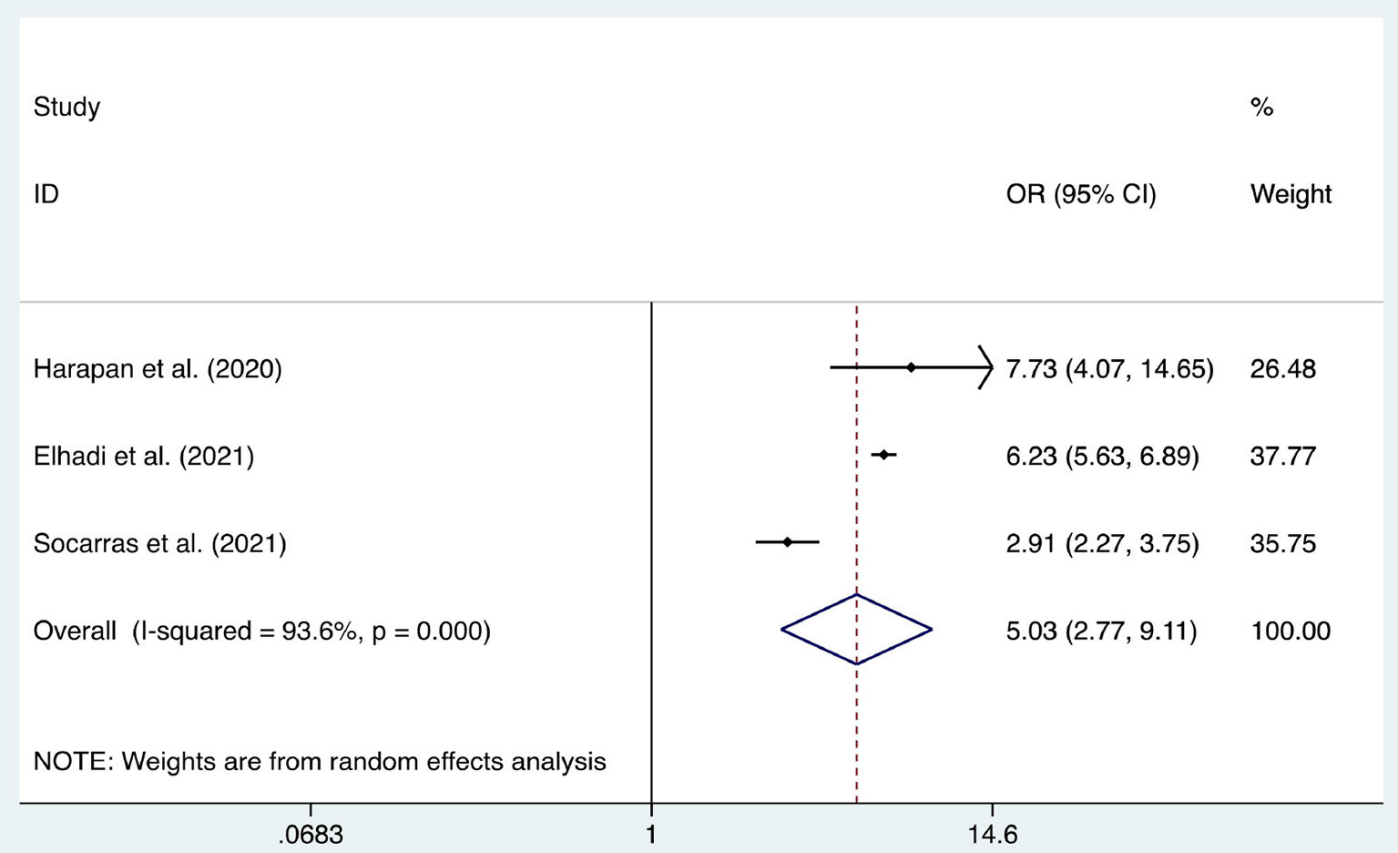
Forest plot of the acceptance of healthcare workers of coronavirus disease 2019 vaccines with different effectiveness (bounded by 70%).

**Figure 7:**
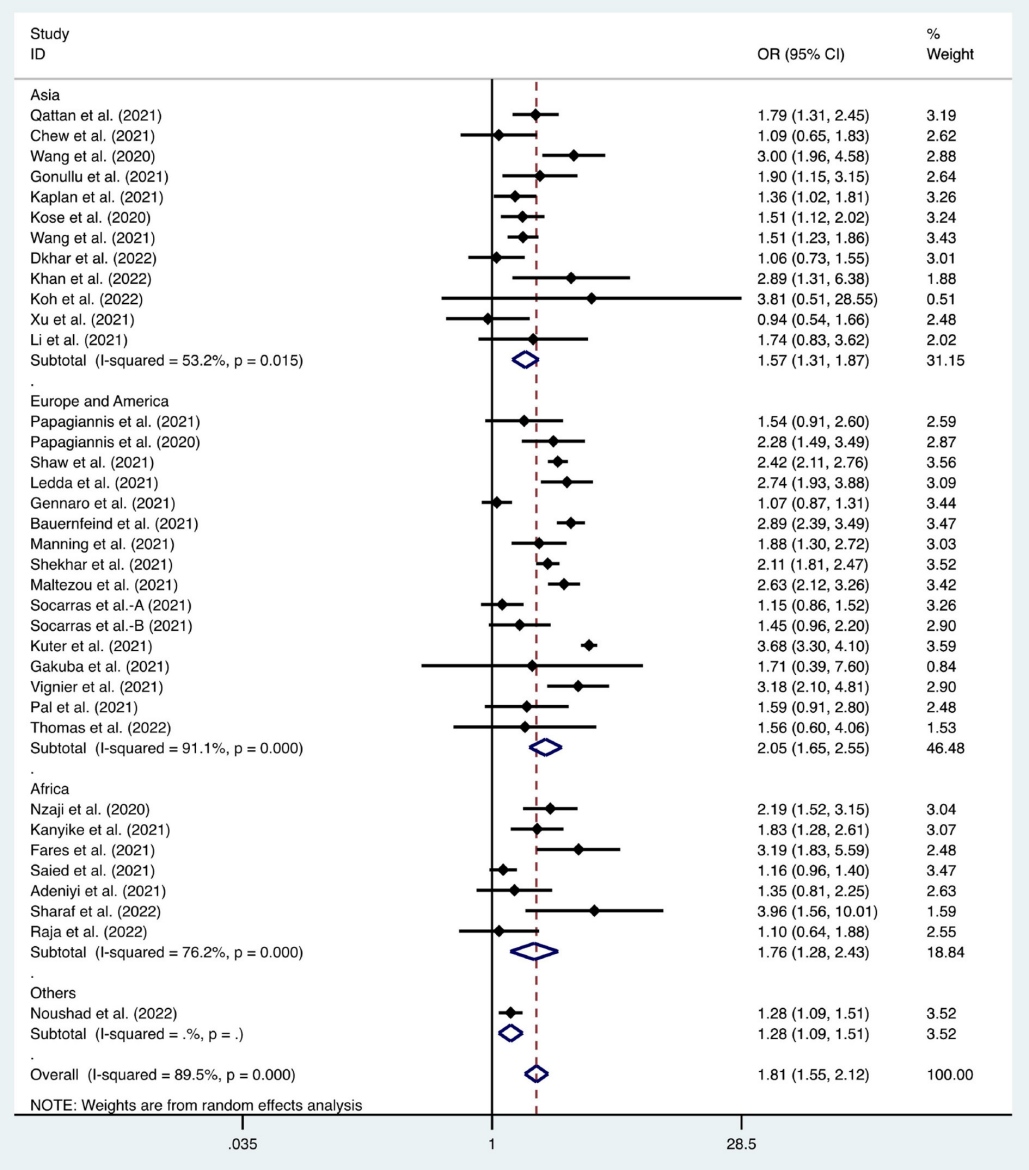
Forest plot of the effect of gender on the willingness of healthcare workers to receive coronavirus disease 2019 vaccines.

**Figure 8:**
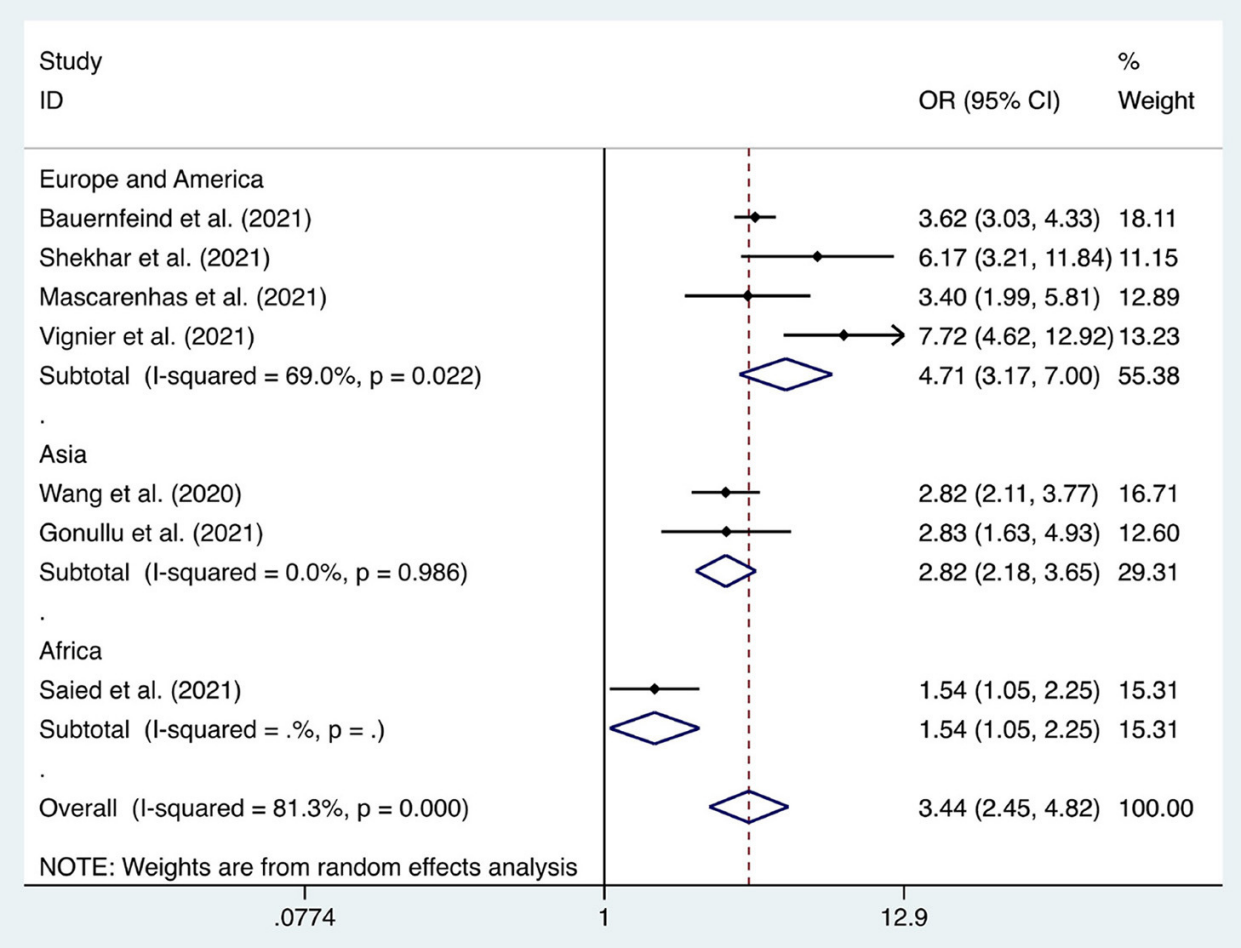
Forest plot of the acceptance of seasonal influenza vaccines by healthcare workers (2019–2020).

**Figure 9:**
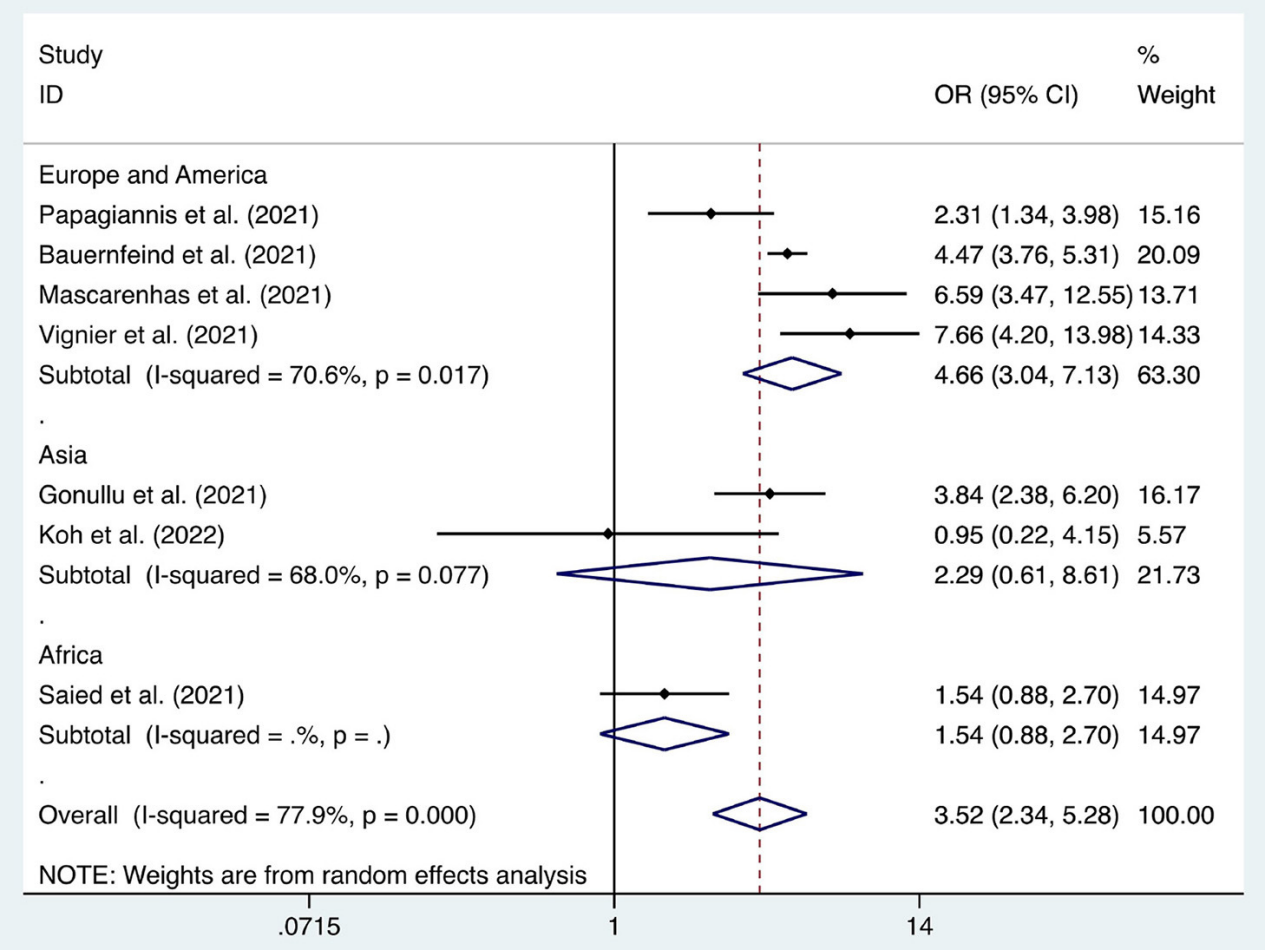
Forest plot of the acceptance of seasonal influenza vaccines by healthcare workers (2020–2021).

**Figure 10:**
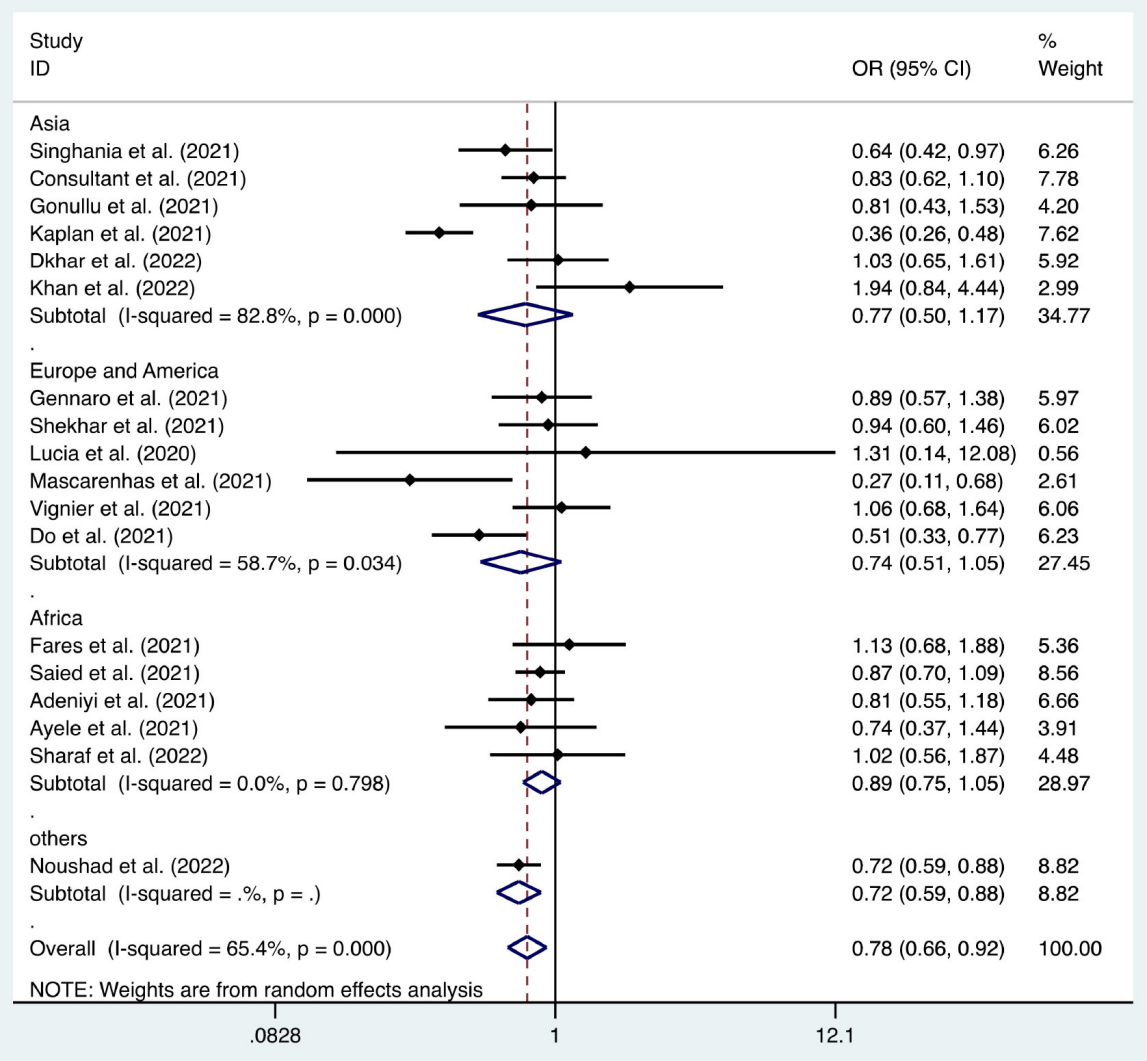
Forest plot of the relationship between healthcare workers' acceptance of the coronavirus disease 2019 vaccination and the infection rate of severe acute respiratory syndrome coronavirus 2.

Seventy-one articles were used to study the acceptance of HCWs about COVID-19 vaccines, which showed that a willingness to undergo COVID-19 vaccination was observed in 66% (95% CI: 0.61–0.67, *I*^2^ = 99.7%, Figure 2) of HCWs. A recent study showed that up to 98% of HCWs in Uganda were willing to be vaccinated against COVID-19 (72). However, through subgroup analysis, we found that only 56% (95% CI: 0.42–0.70, *I*^2^ = 99.8%, Figure 2) of HCWs in African countries were willing to receive COVID-19 vaccination, which was lower than that in Asian (ratio = 0.66, 95% CI: 0.56–0.76, *I*^2^ = 99.8%, Figure 2) and European & American countries (ratio = 0.70, 95% CI: 0.64–0.75, *I*^2^ = 99.5%, Figure 2).

Six articles were used to study the acceptance of HCWs about compulsory vaccination, showing that the proportion of HCWs who agreed with this was 59% (95% CI: 0.46–0.72, *I*^2^ = 98.9%,s Figure 3). We analyzed 24 articles to examine the variance in willingness to take the COVID-19 vaccine between doctors and nurses, and the results indicated that doctors showed a higher willingness to receive COVID-19 vaccination than nurses (OR = 2.22, 95% CI: 1.71–2.89, *I*^2^ = 91.9%, *p* < 0.001, Figure 4). Nine articles were studied to compare the willingness of HCWs and non-HCWs to receive COVID-19 vaccination, and it was found that the willingness of HCWs was greatly increased compared to that of non-HCWs (OR = 1.91, 95% CI: 1.16–3.12, *I*^2^ = 97.0%, *p* = 0.01, Figure 5). Additionally, by analyzing three other articles, we found that with an increased effectiveness of the vaccines in preventing COVID-19 (bounded by 70%), the willingness of HCWs to receive the vaccination also rose accordingly (OR = 5.03, 95% CI: 2.77–9.11, *I*^2^ = 93.6%, *p* < 0.001, Figure 6). The research revealed that male members of HCWs showed a higher willingness to be vaccinated (OR = 1.81, 95% CI: 1.55–2.12, *I*^2^ = 89.5%, *p* < 0.001, Figure 7). The HCWs with a higher acceptance of COVID-19 vaccines were more inclined to receive seasonal influenza vaccines in 2019–2020 (OR = 3.44, 95% CI: 2.45–4.82, *I*^2^ = 81.3%, *p* < 0.001, Figure 8) and 2020–2021 (OR = 3.52, 95% CI: 2.34–5.28, *I*^2^ = 77.9%, *p* < 0.001, Figure 9). Furthermore, the rate of SARS-CoV-2 infection among HCWs willing to be vaccinated was significantly lower than that among HCWs who showed hesitancy (OR = 0.78, 95% CI: 0.66–0.92, *I*^2^ = 65.4%, *p* < 0.001, Figure 10).

Nine articles were used to study the differences between the willingness of HCWs to receive COVID-19 vaccination and the 2020–2021 seasonal influenza vaccines (OR = 1.71, 95% CI: 0.83–3.52, *I*^2^ = 98.9%, *p* = 0.145, Supplementary Figure 1). Seven articles were used to study the impact of the COVID-19 epidemic on seasonal influenza vaccination (2019–2020 and 2020–2021) (OR = 1.43, 95% CI: 0.81–2.53, *I*^2^ = 98.2%, *p* = 0.214, Supplementary Figure 2), and no significant difference was observed in either study.

Some studies have shown that elderly HCWs are more willing to be inoculated with COVID-19 vaccines (20, 28, 51). Nevertheless, a study from Zhejiang Province, China, showed that a large number of HCWs aged over 50 years experienced SARS in 2003, influenza A (H1N1) in 2009 and avian influenza A (H7N9) in 2013. With the exception of H1N1, the other two were well contained without introducing vaccination, so some people would inevitably assume that vaccination against COVID-19 was probably not necessary (54). Married HCWs were remarkably more willing to be vaccinated for the protection of their families (47). However, a study from Uganda came to the opposite conclusion. Their study revealed that single HCWs showed a higher acceptance of COVID-19 vaccines (15). To solve similar contradictions, we compared the characteristics of HCWs from two groups, one with HCWs who were willing to be inoculated with COVID-19 vaccines and another with those who were not. The results showed that age [(OR = 0.91, 95% CI: 0.75–1.12, *I*^2^ = 89.3%, *p* = 0.145, Supplementary Figure 3) and (OR = 0.85, 95% CI: 0.63–1.14, *I*^2^ = 90.1%, *p* = 0.288, Supplementary Figure 4)], education level (OR = 0.81, 95% CI: 0.54–1.22, *I*^2^ = 94.2%, *p* = 0.315, Supplementary Figure 5), marriage status (OR = 0.96, 95% CI: 0.75–1.23, *I*^2^ = 71.9%, *p* = 0.758, Supplementary Figure 6), close contact with COVID-19 patients (OR = 1.01, 95% CI: 0.77–1.32, *I*^2^ = 94.1%, *p* = 0.959, Supplementary Figure 7), and chronic diseases (OR = 1.19, 95% CI: 0.90–1.59, *I*^2^ = 90.6%, *p* = 0.222, Supplementary Figure 8) did not significantly affect the acceptance of COVID-19 vaccines by HCWs. The factors associated with COVID-19 vaccine acceptance of HCWs are listed in Table 3.

**Table 3:** The factors associated with COVID-19 vaccine acceptance of HCWs.

**Variables**	**Included studies**	**OR**	**95% CI**	***P-*value**	***I^2^***
Occupation (doctors and nurses)	[14, 16, 17, 20, 23, 24, 30, 32, 33, 35, 39, 44, 46–48, 50, 54, 58, 59, 61, 63, 69, 73, 78]	2.22	1.71–2.89	<0.001	91.90%
Occupation (HCWs and non-HCWs)	[13, 19, 40, 50, 52, 55, 65, 74, 79]	1.91	1.16–3.12	0.01	97.00%
Vaccine effectiveness	[13, 39, 42]	5.03	2.77–9.11	<0.001	93.60%
Gender	[10–12, 15–18, 20, 22, 23, 25–27, 33, 37, 41–43, 47–49, 53, 54, 56, 57, 58, 60, 62, 64, 65–67, 71, 76, 78]	1.81	1.55–2.12	<0.001	89.50%
Seasonal influenza vaccines (2019–2020)	[6, 23, 27, 37, 41, 49, 60]	3.44	2.45–4.82	<0.001	81.30%
Seasonal influenza vaccines (2020–2021)	[6, 11, 23, 41, 49, 60, 64]	3.52	2.34–5.28	<0.001	77.90%
SARS-CoV-2 infection	[6, 14, 22, 25, 27, 30, 31, 41, 47, 49, 56, 57, 58, 59, 60, 61, 62, 65]	0.78	0.66–0.92	<0.001	65.40%
Age (bounded by 40)	[10, 12, 20, 22, 26, 27, 33, 37, 43, 47, 59, 65, 71, 78]	0.91	0.75–1.12	0.145	89.30%
Age (bounded by 50)	[10, 20, 22, 26, 27, 30, 33, 37, 46, 47, 54, 56, 60, 65, 78]	0.85	0.63–1.14	0.288	90.10%
Education level	[16, 23, 25, 27, 43, 54, 58, 63, 67, 76, 78]	0.81	0.54–1.22	0.315	94.20%
Marriage status	[10, 12, 15, 16, 46, 47, 57, 59, 78]	0.96	0.75–1.23	0.758	71.90%
Close contact with COVID-19 patients	[10, 14, 18, 23, 25, 27, 33, 37, 41, 46, 47, 54, 57, 58, 64, 65, 67, 71]	1.01	0.77–1.32	0.959	94.10%
Chronic diseases	[10, 16, 20, 27, 33, 35, 37, 41, 46, 47, 48, 56, 58, 59, 65]	1.19	0.90–1.59	0.222	90.60%

HCWs, Healthcare workers; COVID-19, coronavirus disease 2019; SARS-CoV-2, severe acute respiratory syndrome coronavirus 2; OR, odds ratio; CI, confidence interval.

## Discussion

The vaccine is metaphorically known as the “seatbelt against the disease,” which can effectively protect people against infectious diseases at the lowest cost (79). In improving public health, vaccination functions as one of the most important advances. It successfully promoted the elimination of smallpox worldwide and the control of numerous infectious diseases (e.g., rubella, diphtheria, polio) (80). It is estimated that approximately two to three million deaths can be avoided each year by vaccination (81). Despite this, public distrust of vaccines is widespread. The most typical example is the boycott of polio vaccination in northern Nigeria in 2003–2004 (82). Frontline HCWs are frequently and closely exposed to highly contagious patients with COVID-19, posing them at highly increased risk of infection and transmission. Therefore, they became the primary concern of authorities around the world when they formulated COVID-19 vaccination policies (19). Our research showed that approximately 66% of HCWs were willing to receive COVID-19 vaccines, which might vary among different regions. A report showed that only 21% of HCWs in Egypt held a positive attitude toward COVID-19 vaccines (25). A survey on the Asia Pacific region showed that the acceptance of COVID-19 vaccines by HCWs in six countries, including China and India, approached nearly 96% (16). Since a compulsory vaccination program can effectively increase the overall vaccination coverage rate (83), we considered the views of HCWs on this measure, and the results showed that approximately 59% of HCWs agreed with it. We additionally studied the impact of the COVID-19 epidemic on vaccination against seasonal influenza and the association between the two. The prior experience gained from seasonal influenza vaccination provides a reference and guidance for COVID-19 vaccination. It was noticed that the COVID-19 epidemic did not significantly affect the seasonal influenza vaccination of HCWs; however, interestingly, HCWs who showed a stronger intention to vaccinate against COVID-19 were more likely to receive seasonal influenza vaccination. The experience of influenza vaccination has been known as one of the drivers of accepting COVID-19 vaccines (84). It was also discovered that when the effectiveness of the vaccines changed, the acceptance of the vaccines by HCWs varied accordingly. In our meta-analysis, HCWs demonstrated a higher acceptance of COVID-19 vaccines than non-HCWs. Even in HCWs, the acceptance of COVID-19 vaccines varied among individuals with different occupations. In particular, doctors showed significantly higher acceptance of COVID-19 vaccines than nurses.

It was comparatively found that males were more willing to be vaccinated against COVID-19 than females among HCWs. The higher willingness of males to receive COVID-19 vaccination could be attributed to social and cultural differences and males' risk-taking tendency (85). Some reports indicated that males were at a higher risk of experiencing COVID-19 complications, infections, and even deaths (86). Our study showed that HCWs willing to be vaccinated against COVID-19 experienced a lower risk of infection, probably owing to a high level of protection awareness among them.

The HCWs who remained skeptical about vaccination against COVID-19 were mainly concerned about the efficacy and safety of the vaccines due to the short duration of vaccine development (18, 22, 25, 33). The rapid spread of misleading information about COVID-19 vaccines on various media platforms has aggravated HCWs' doubts about them (10). Since the acceptance of HCWs directly affects the trust of non-HCWs in COVID-19 vaccines, it is necessary to boost their confidence.

## Limitations

The data were collected from various countries and regions in the world. Due to the different severities of the outbreak, various prevention and control measures, and cultural and cognitive differences, the heterogeneity of our results was generally high.

People's intention to vaccinate against COVID-19 will change with the epidemic situation (37). Even in the same region, there will be certain variations in the statistical data at different periods.

## Conclusions

Our research revealed that a considerable percentage of HCWs remained skeptical about COVID-19 vaccines. Five factors: occupation, gender, vaccine effectiveness, seasonal influenza vaccines, and SARS-CoV-2 infection; significantly affected the willingness of HCWs to be vaccinated against COVID-19. Herein, it is essential to boost the confidence of HCWs in COVID-19 vaccines for the containment of the epidemic.

## Data availability statement

The original contributions presented in the study are included in the article/Supplementary material, further inquiries can be directed to the corresponding author.

## Author contributions

Project administration and data curation: JL. Writing-original draft preparation: LW and YW. Writing-review and editing: XC and XL. Software: YY. All authors read and approved the final manuscript.

## Conflict of interest

The authors declare that the research was conducted in the absence of any commercial or financial relationships that could be construed as a potential conflict of interest.

## Publisher's note

All claims expressed in this article are solely those of the authors and do not necessarily represent those of their affiliated organizations, or those of the publisher, the editors and the reviewers. Any product that may be evaluated in this article, or claim that may be made by its manufacturer, is not guaranteed or endorsed by the publisher.
