# Supplementary material for: Acceptance of coronavirus disease 2019 (COVID-19) vaccines among healthcare workers: A meta-analysis
Source: Front Public Health. 2022 Sep 16;10:881903. doi: 10.3389/fpubh.2022.881903 (PMC9525162; doi:10.3389/fpubh.2022.881903)
Supplement: Supplementary file 1 [file Data_Sheet_1.zip › Legends to Supplementary Figures.docx]

**Supplementary Figure 1:** Forest plot of the difference in healthcare workers’ willingness to receive coronavirus disease 2019 vaccination and the 2020-2021 seasonal influenza vaccination.

**Supplementary Figure 2:** Forest plot of the impact of the coronavirus disease 2019 epidemic on seasonal influenza vaccination (2019–2020 and 2020–2021).

**Supplementary Figure 3:** Forest plot of the acceptance of the coronavirus disease 2019 vaccines by healthcare workers of different ages (bounded by 40).

**Supplementary Figure 4:** Forest plot of the acceptance of coronavirus disease 2019 vaccines by healthcare workers of different ages (bounded by 50).

**Supplementary Figure 5:** Forest plot of the acceptance of healthcare workers with different education levels about coronavirus disease 2019 vaccines.

**Supplementary Figure 6:** Forest plot of the acceptance of healthcare workers with different marital statuses about coronavirus disease 2019 vaccines.

**Supplementary Figure 7:** Forest plot of the effect of close contact with coronavirus disease 2019 patients on the willingness of healthcare workers to receive coronavirus disease 2019 vaccines.

**Supplementary Figure 8:** Forest plot of the effect of chronic diseases on the willingness of healthcare workers to receive coronavirus disease 2019 vaccines.
