# Supplementary figures and images for: Acceptance of coronavirus disease 2019 (COVID-19) vaccines among healthcare workers: A meta-analysis
Source: Front Public Health. 2022 Sep 16;10:881903. doi: 10.3389/fpubh.2022.881903 (PMC9525162; doi:10.3389/fpubh.2022.881903)

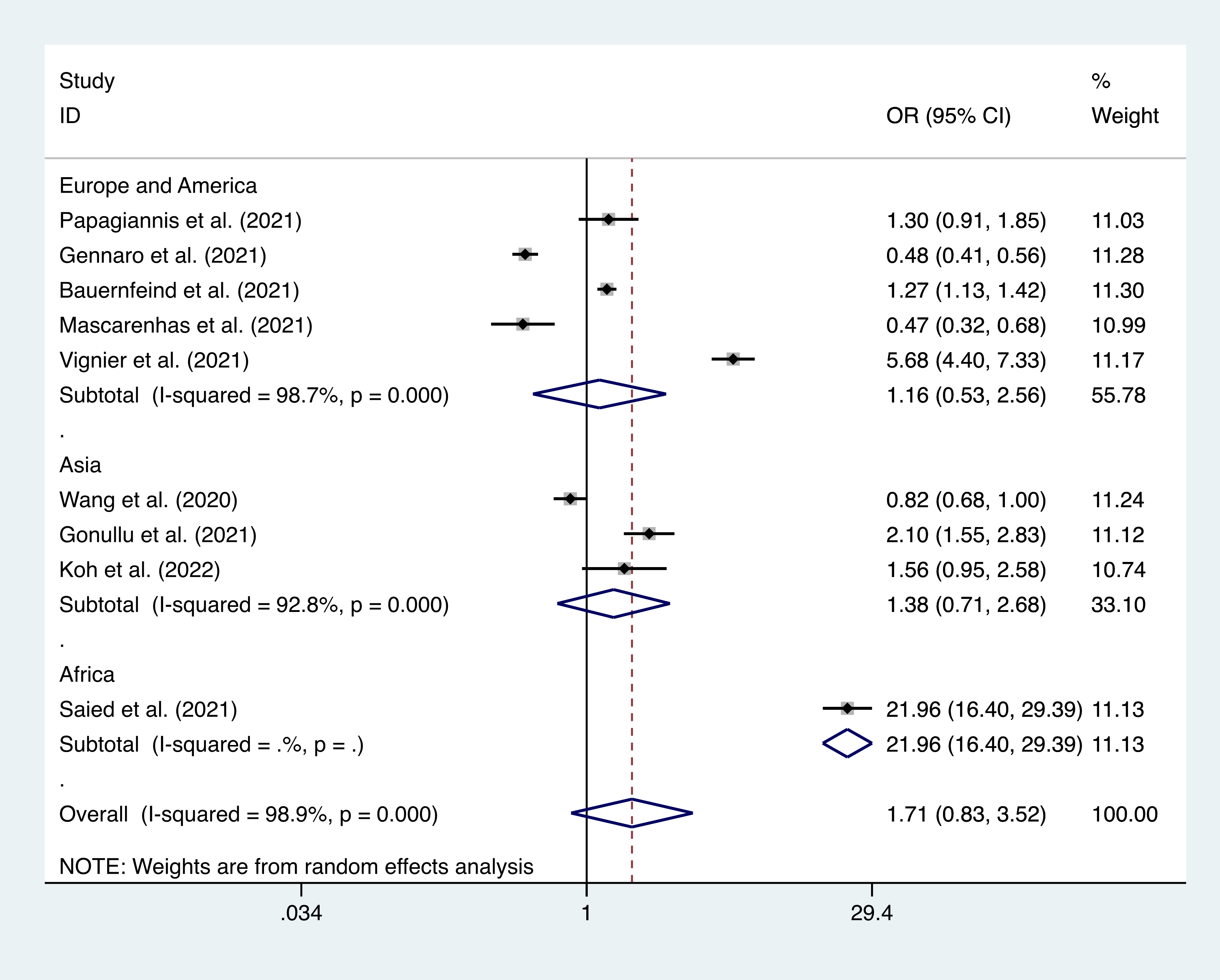

Supplement: Supplementary file 1 [file Data_Sheet_1.zip › Supplementary Figure 1.jpg]

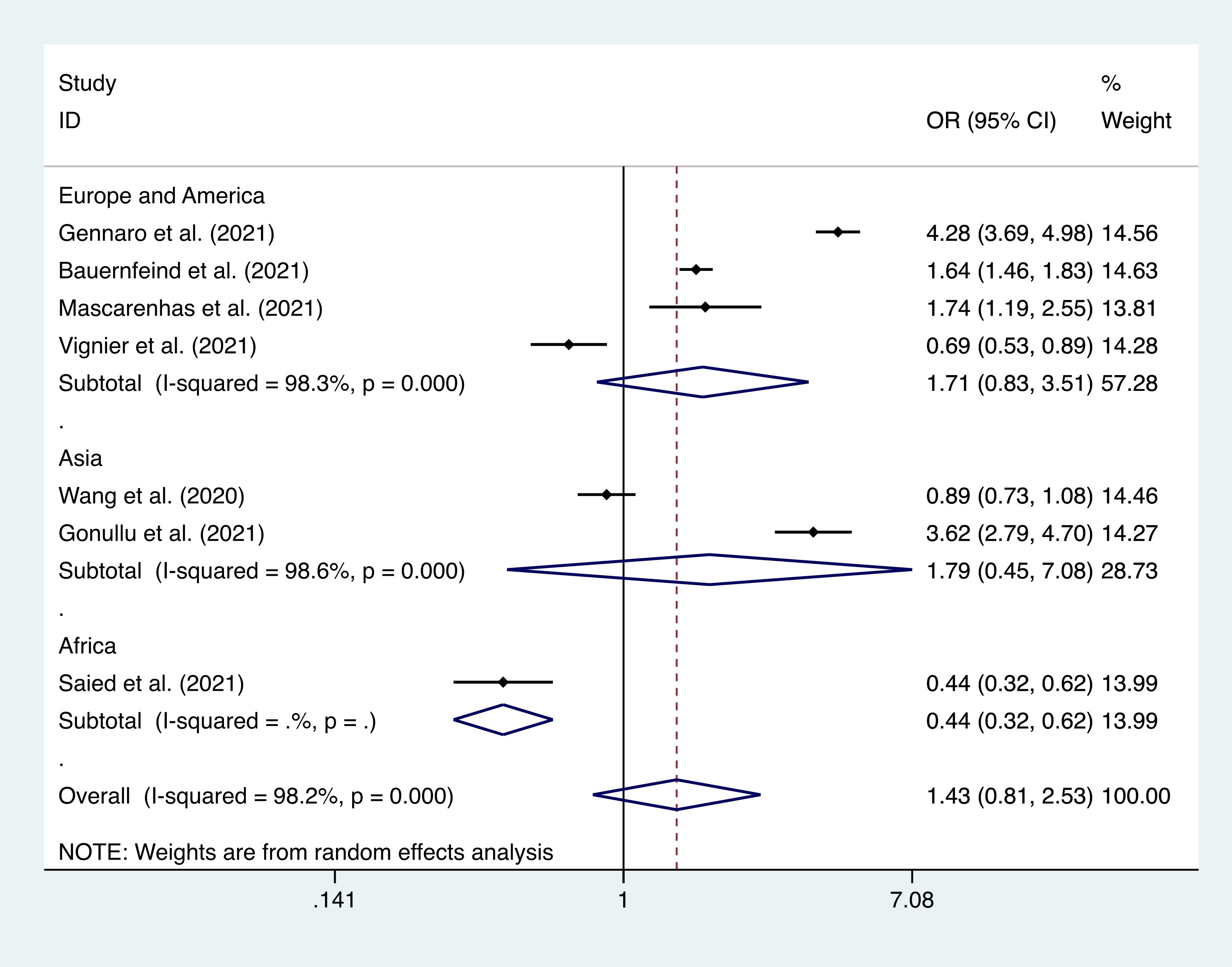

Supplement: Supplementary file 1 [file Data_Sheet_1.zip › Supplementary Figure 2.jpg]

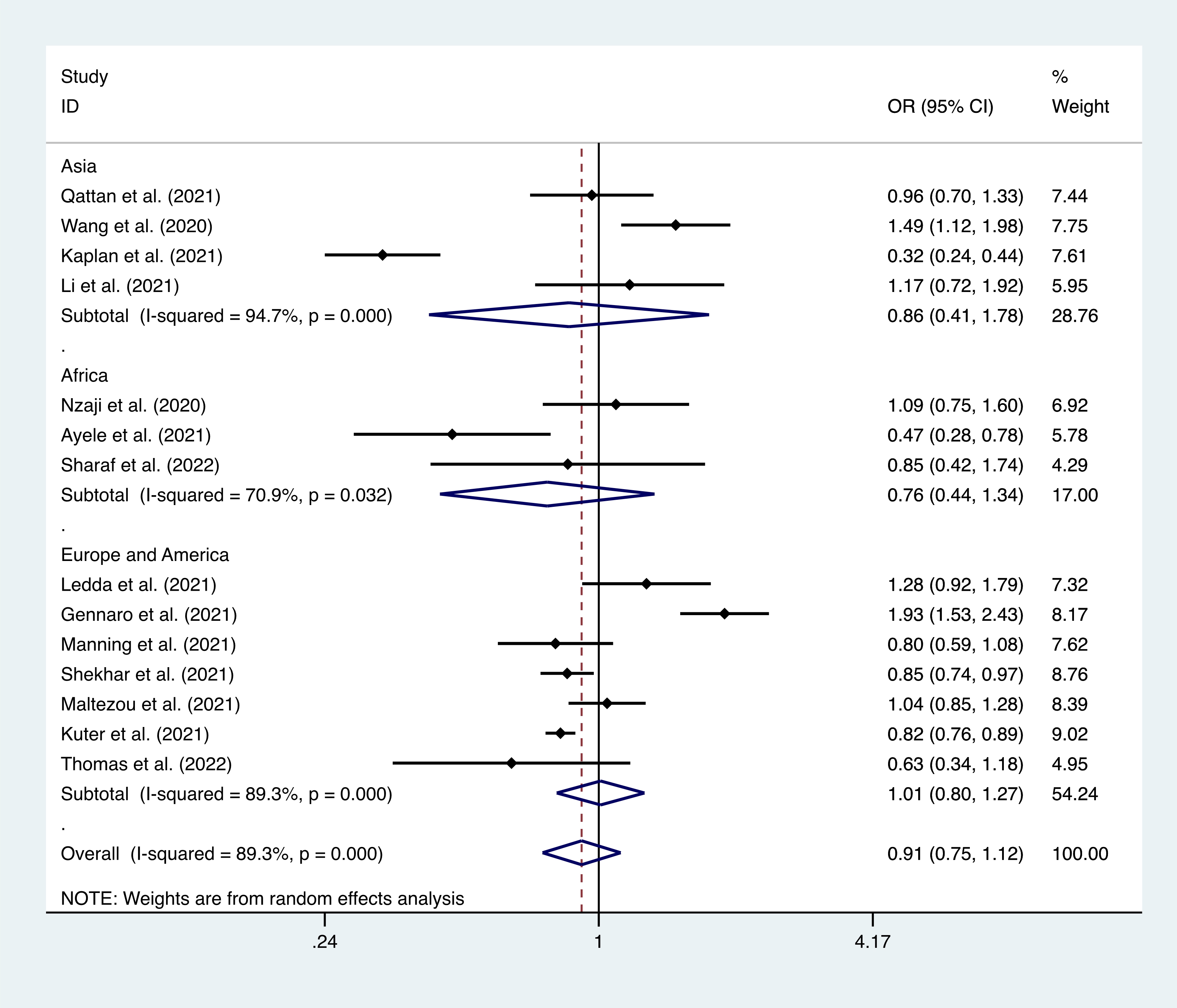

Supplement: Supplementary file 1 [file Data_Sheet_1.zip › Supplementary Figure 3.jpg]

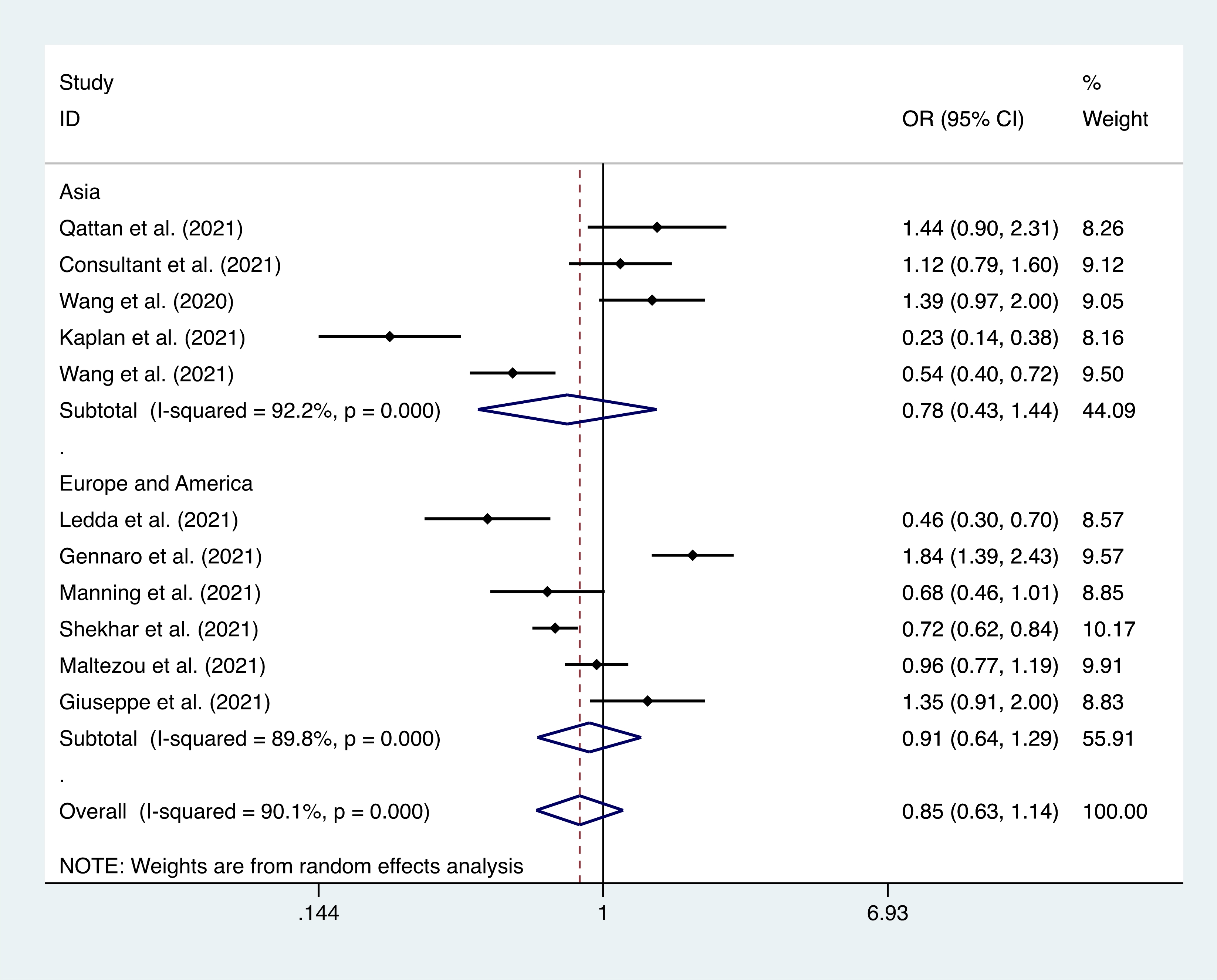

Supplement: Supplementary file 1 [file Data_Sheet_1.zip › Supplementary Figure 4.jpg]

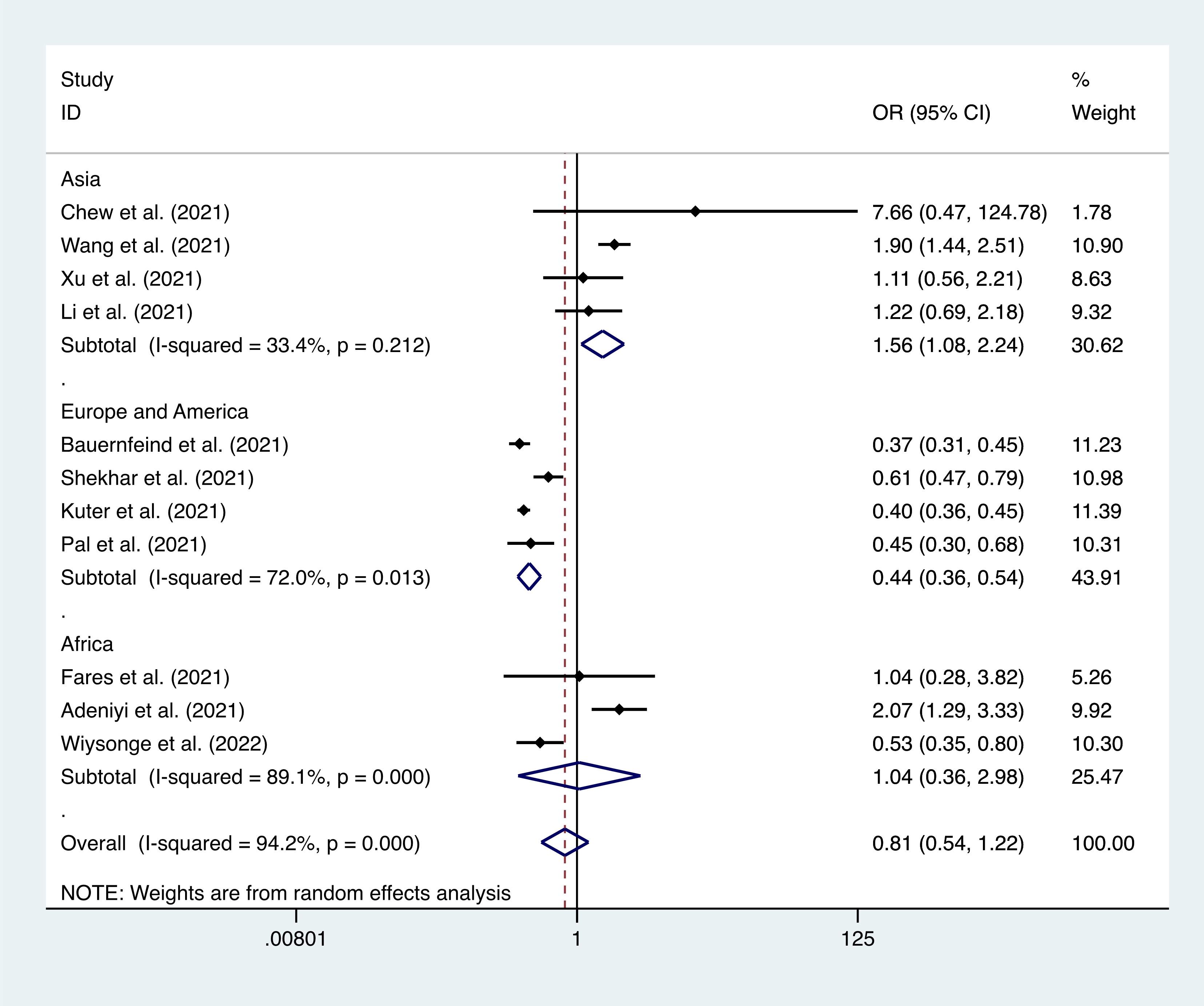

Supplement: Supplementary file 1 [file Data_Sheet_1.zip › Supplementary Figure 5.jpg]

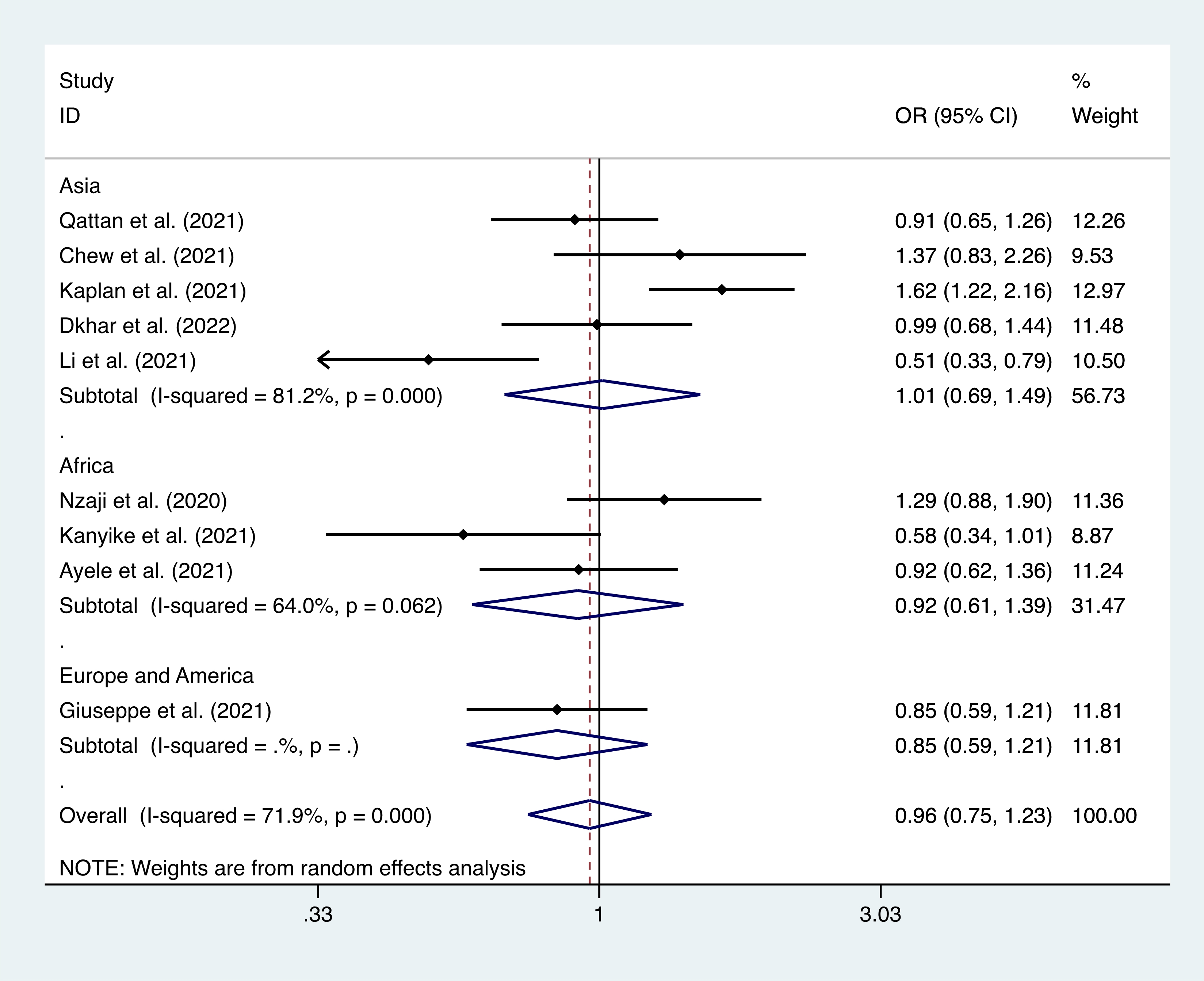

Supplement: Supplementary file 1 [file Data_Sheet_1.zip › Supplementary Figure 6.jpg]

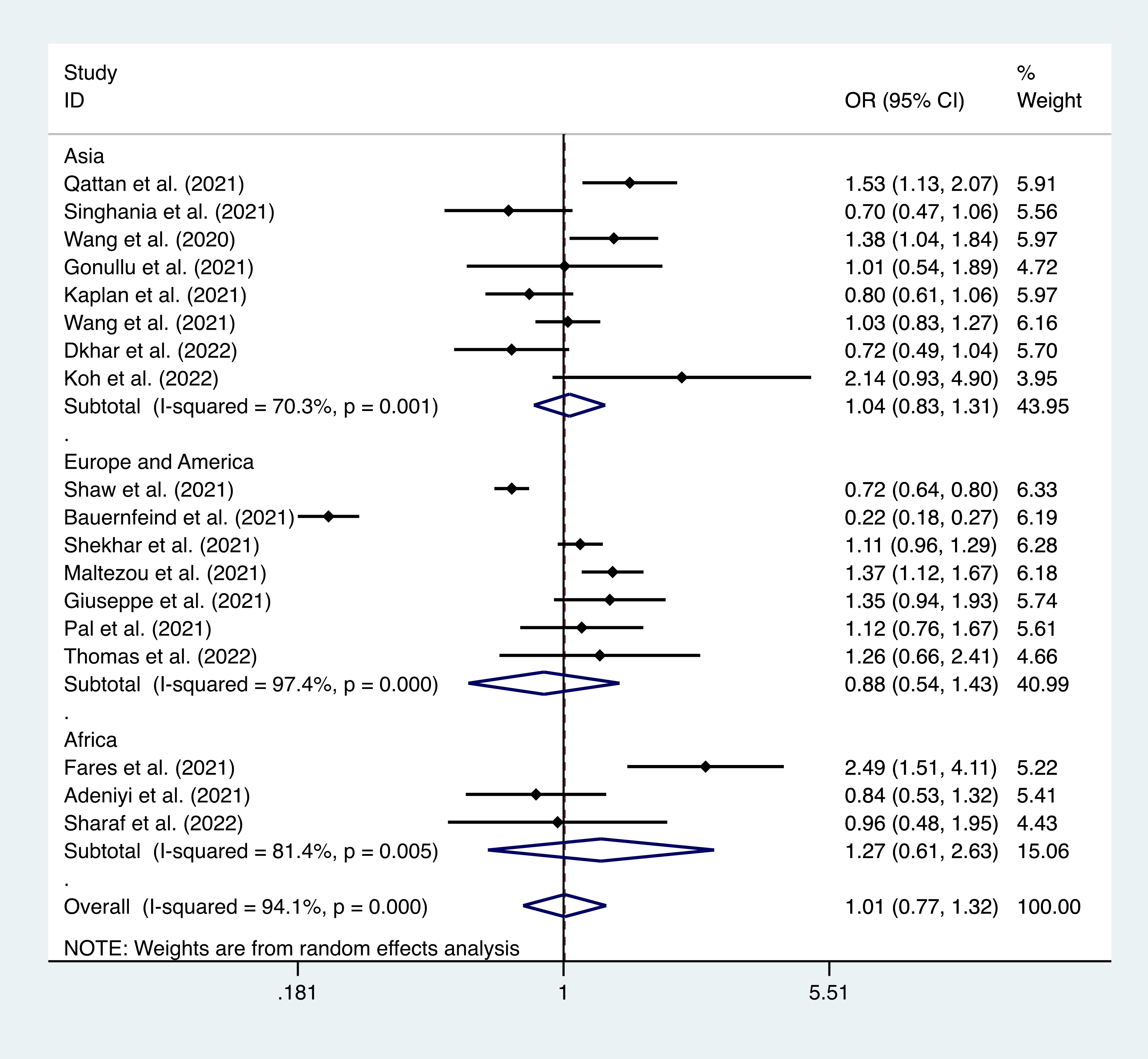

Supplement: Supplementary file 1 [file Data_Sheet_1.zip › Supplementary Figure 7.jpg]

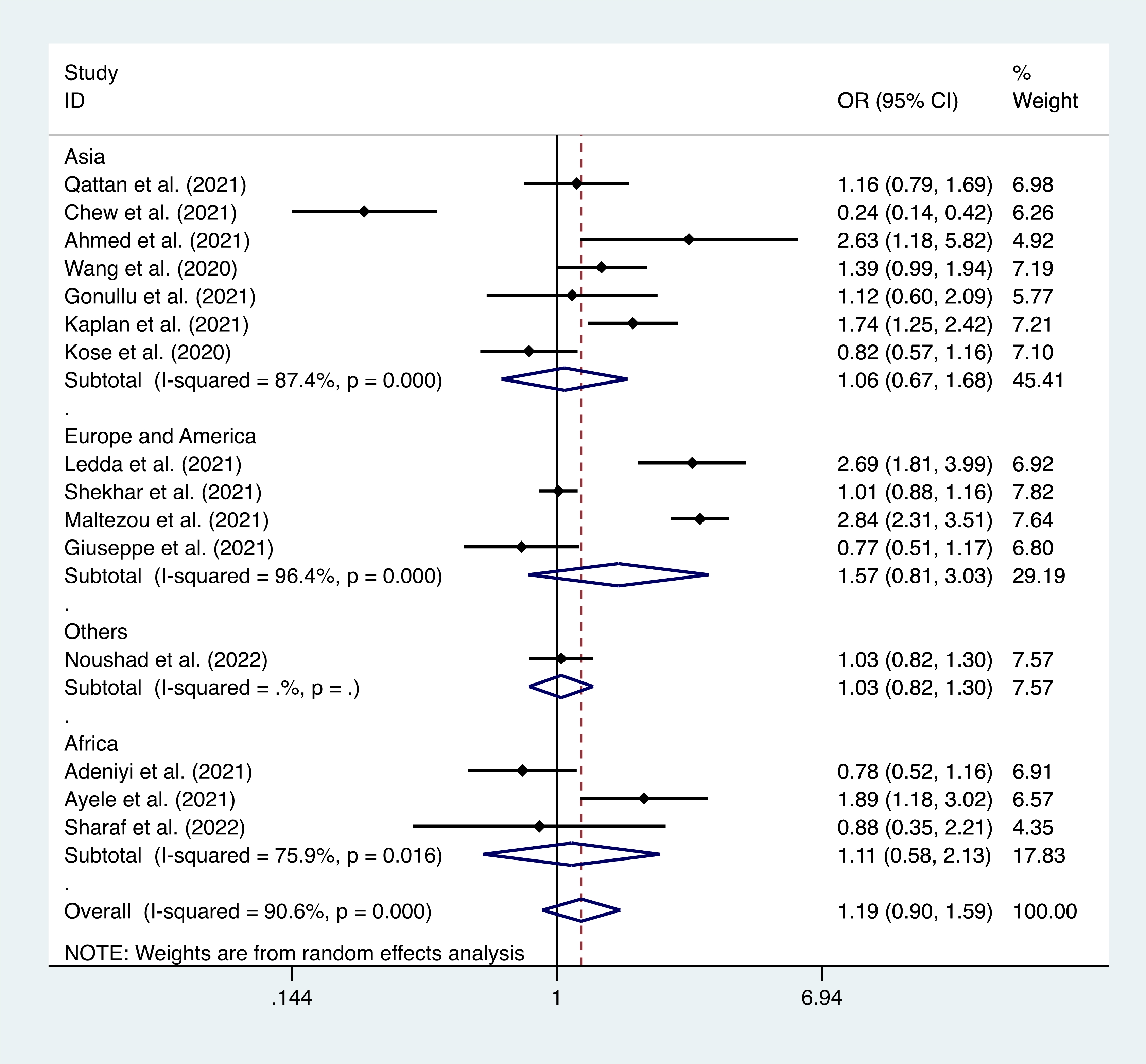

Supplement: Supplementary file 1 [file Data_Sheet_1.zip › Supplementary Figure 8.jpg]
