# Supplementary material for: Acceptance of coronavirus disease 2019 (COVID-19) vaccines among healthcare workers: A meta-analysis
Source: Front Public Health. 2022 Sep 16;10:881903. doi: 10.3389/fpubh.2022.881903 (PMC9525162; doi:10.3389/fpubh.2022.881903)
Supplement: Supplementary file 1 [file Data_Sheet_1.zip › Supplementary File 1.doc]

**In Pubmed, the retrieval strategy is:**

((("COVID-19 Vaccines"[Mesh]) OR ((((((((((((((((((((((((((((((((((((((((((((((((((((COVID 19 Vaccines[Title/Abstract]) OR (Vaccines, COVID-19[Title/Abstract])) OR (COVID-19 Virus Vaccines[Title/Abstract])) OR (COVID 19 Virus Vaccines[Title/Abstract])) OR (Vaccines, COVID-19 Virus[Title/Abstract])) OR (Virus Vaccines, COVID-19[Title/Abstract])) OR (COVID-19 Virus Vaccine[Title/Abstract])) OR (COVID 19 Virus Vaccine[Title/Abstract])) OR (Vaccine, COVID-19 Virus[Title/Abstract])) OR (Virus Vaccine, COVID-19[Title/Abstract])) OR (COVID19 Virus Vaccines[Title/Abstract])) OR (Vaccines, COVID19 Virus[Title/Abstract])) OR (Virus Vaccines, COVID19[Title/Abstract])) OR (COVID19 Virus Vaccine[Title/Abstract])) OR (Vaccine, COVID19 Virus[Title/Abstract])) OR (Virus Vaccine, COVID19[Title/Abstract])) OR (COVID19 Vaccines[Title/Abstract])) OR (Vaccines, COVID19[Title/Abstract])) OR (Vaccine, COVID19[Title/Abstract])) OR (SARS-CoV-2 Vaccines[Title/Abstract])) OR (SARS CoV 2 Vaccines[Title/Abstract])) OR (Vaccines, SARS-CoV-2[Title/Abstract])) OR (SARS-CoV-2 Vaccine[Title/Abstract])) OR (SARS CoV 2 Vaccine[Title/Abstract])) OR (Vaccine, SARS-CoV-2[Title/Abstract])) OR (SARS2 Vaccines[Title/Abstract])) OR (Vaccines, SARS2[Title/Abstract])) OR (SARS2 Vaccine[Title/Abstract])) OR (Vaccine, SARS2[Title/Abstract])) OR (Coronavirus Disease 2019 Vaccines[Title/Abstract])) OR (Coronavirus Disease 2019 Vaccine[Title/Abstract])) OR (Coronavirus Disease 2019 Virus Vaccine[Title/Abstract])) OR (Coronavirus Disease 2019 Virus Vaccines[Title/Abstract])) OR (Coronavirus Disease-19 Vaccines[Title/Abstract])) OR (Coronavirus Disease 19 Vaccines[Title/Abstract])) OR (Vaccines, Coronavirus Disease-19[Title/Abstract])) OR (Coronavirus Disease-19 Vaccine[Title/Abstract])) OR (Coronavirus Disease 19 Vaccine[Title/Abstract])) OR (Vaccine, Coronavirus Disease-19[Title/Abstract])) OR (COVID 19 Vaccine[Title/Abstract])) OR (Vaccine, COVID 19[Title/Abstract])) OR (2019-nCoV Vaccine[Title/Abstract])) OR (2019 nCoV Vaccine[Title/Abstract])) OR (Vaccine, 2019-nCoV[Title/Abstract])) OR (2019 Novel Coronavirus Vaccines[Title/Abstract])) OR (2019 Novel Coronavirus Vaccine[Title/Abstract])) OR (2019-nCoV Vaccines[Title/Abstract])) OR (2019 nCoV Vaccines[Title/Abstract])) OR (Vaccines, 2019-nCoV[Title/Abstract])) OR (COVID-19 Vaccine[Title/Abstract])) OR (Vaccine, COVID-19[Title/Abstract])) OR (SARS Coronavirus 2 Vaccines[Title/Abstract]))) AND ((((acceptability[Title/Abstract]) OR (attitude[Title/Abstract])) OR (acceptance[Title/Abstract])) OR (willingness[Title/Abstract]))) AND (((((((((((healthcare personnel[Title/Abstract]) OR (healthcare worker[Title/Abstract])) OR (healthcare workers[Title/Abstract])) OR (health professionals[Title/Abstract])) OR (medical services workers[Title/Abstract])) OR ((medical workers[Title/Abstract])) OR (dental[Title/Abstract])) OR (nurses[Title/Abstract])) OR (nurse[Title/Abstract])) OR (pediatricians[Title/Abstract])) OR (doctors[Title/Abstract])).
