# Supplementary material for: Acceptance of coronavirus disease 2019 (COVID-19) vaccines among healthcare workers: A meta-analysis
Source: Front Public Health. 2022 Sep 16;10:881903. doi: 10.3389/fpubh.2022.881903 (PMC9525162; doi:10.3389/fpubh.2022.881903)
Supplement: Supplementary file 1 [file Data_Sheet_1.zip › Supplementary File 2.doc]

1. Egger’s publication bias plot of the difference in healthcare workers’ willingness to receive coronavirus disease 2019 vaccination and the 2020-2021 seasonal influenza vaccination (p = 0.375).

2. Egger’s publication bias plot of the impact of the coronavirus disease 2019 epidemic on seasonal influenza vaccination (2019–2020 and 2020–2021) (p = 0.456).

3. Egger’s publication bias plot of the difference in the willingness between doctors and nurses to receive coronavirus disease 2019 vaccines (p = 0.111).

4. Egger’s publication bias plot of the willingness of healthcare workers (HCWs) and non-HCWs to receive coronavirus disease 2019 vaccines (p = 0.151).

5. Egger’s publication bias plot of the acceptance of healthcare workers of coronavirus disease 2019 vaccines with different effectiveness (bounded by 70%) (p = 0.768).

6. Egger’s publication bias plot of the acceptance of the coronavirus disease 2019 vaccines by healthcare workers of different ages (bounded by 40) (p = 0.680).

7. Egger’s publication bias plot of the acceptance of coronavirus disease 2019 vaccines by healthcare workers of different ages (bounded by 50) (p = 0.959).

8. Egger’s publication bias plot of the acceptance of healthcare workers with different education levels about coronavirus disease 2019 vaccines (p = 0.049).

9. Egger’s publication bias plot of the acceptance of healthcare workers with different marital statuses about coronavirus disease 2019 vaccines (p = 0.125).

10. Egger’s publication bias plot of the effect of close contact with coronavirus disease 2019 patients on the willingness of healthcare workers to receive coronavirus disease 2019 vaccines (p = 0.279).

11. Egger’s publication bias plot of the effect of chronic diseases on the willingness of healthcare workers to receive coronavirus disease 2019 vaccines (p = 0.805).

12. Egger’s publication bias plot of the effect of gender on the willingness of healthcare workers to receive coronavirus disease 2019 vaccines (p = 0.118).

13. Egger’s publication bias plot of the acceptance of seasonal influenza vaccines by healthcare workers (2019–2020) (p = 0.835).

14. Egger’s publication bias plot of the acceptance of seasonal influenza vaccines by healthcare workers (2020–2021) (p = 0.382).

15. Egger’s publication bias plot of the relationship between healthcare workers' acceptance of the coronavirus disease 2019 vaccination and the infection rate of severe acute respiratory syndrome coronavirus 2 (p = 0.524).
