# Supplementary material for: Acceptance of coronavirus disease 2019 (COVID-19) vaccines among healthcare workers: A meta-analysis
Source: Front Public Health. 2022 Sep 16;10:881903. doi: 10.3389/fpubh.2022.881903 (PMC9525162; doi:10.3389/fpubh.2022.881903)
Supplement: Supplementary file 1 [file Data_Sheet_1.zip › Supplementary Table 1.doc]

**Supplementary Table 1 :** Newcastle–Ottawa quality assessment scale.

| Study | Publication year | Selection | Comparability | Outcome | Quality assessment |
| --- | --- | --- | --- | --- | --- |
| Mascarenhas et al. | 2021 | 4 | 1 | 2 | good |
| Qattan et al. | 2021 | 4 | 1 | 2 | good |
| Papagiannis et al. | 2021 | 4 | 1 | 2 | good |
| Nzaji et al. | 2020 | 4 | 1 | 2 | good |
| Harapan et al. | 2020 | 4 | 2 | 3 | good |
| Singhania et al. | 2021 | 4 | 1 | 2 | good |
| Kanyike et al. | 2021 | 4 | 1 | 2 | good |
| Chew et al. | 2021 | 4 | 1 | 2 | good |
| Papagiannis et al. | 2020 | 4 | 1 | 2 | good |
| Shaw et al. | 2021 | 4 | 1 | 2 | good |
| Szmyd et al. | 2021 | 4 | 1 | 2 | good |
| Ledda et al. | 2021 | 4 | 1 | 2 | good |
| Verger et al. | 2021 | 4 | 1 | 2 | good |
| Gennaro et al. | 2021 | 4 | 1 | 2 | good |
| Bauernfeind et al. | 2021 | 4 | 1 | 2 | good |
| Fares et al. | 2021 | 4 | 1 | 2 | good |
| Manning et al. | 2021 | 4 | 1 | 2 | good |
| Shekhar et al. | 2021 | 4 | 1 | 2 | good |
| Maraqa et al. | 2021 | 4 | 1 | 2 | good |
| Lucia et al. | 2020 | 4 | 1 | 2 | good |
| Gadoth et al. | 2021 | 4 | 1 | 2 | good |
| Maltezou et al. | 2021 | 4 | 1 | 2 | good |
| Ahmed et al. | 2021 | 4 | 1 | 2 | good |
| Kwok et al. | 2021 | 4 | 1 | 2 | good |
| Wang et al. | 2020 | 4 | 1 | 2 | good |
| Elhadi et al. | 2021 | 4 | 2 | 3 | good |
| Szmyd et al. | 2021 | 4 | 1 | 2 | good |
| Gonullu et al. | 2021 | 4 | 1 | 2 | good |
| Socarras et al. | 2021 | 4 | 2 | 3 | good |
| Kuter et al. | 2021 | 4 | 1 | 2 | good |
| Yu et al. | 2021 | 4 | 1 | 2 | good |
| Giuseppe et al. | 2021 | 4 | 1 | 2 | good |
| Kaplan et al. | 2021 | 4 | 1 | 2 | good |
| Kose et al. | 2020 | 4 | 1 | 2 | good |
| Saied et al. | 2021 | 4 | 1 | 2 | good |
| Dror et al. | 2020 | 4 | 1 | 2 | good |
| Kukreti et al. | 2021 | 4 | 1 | 2 | good |
| Gakuba et al. | 2021 | 4 | 1 | 2 | good |
| Wang et al. | 2021 | 4 | 1 | 2 | good |
| Yurttas et al. | 2021 | 4 | 1 | 2 | good |
| Noushad et al. | 2022 | 4 | 1 | 3 | good |
| Dkhar et al. | 2022 | 4 | 1 | 3 | good |
| Adeniyi et al. | 2021 | 4 | 1 | 2 | good |
| Ayele et al. | 2021 | 4 | 1 | 2 | good |
| Vignier et al. | 2021 | 4 | 1 | 2 | good |
| Do et al. | 2021 | 4 | 1 | 3 | good |
| Khan et al. | 2022 | 4 | 1 | 2 | good |
| Wiysonge et al. | 2022 | 4 | 1 | 2 | good |
| Koh et al. | 2022 | 4 | 1 | 2 | good |
| Sharaf et al. | 2022 | 4 | 1 | 2 | good |
| Raja et al. | 2022 | 4 | 1 | 2 | good |
| Pal et al. | 2021 | 4 | 1 | 2 | good |
| Hara et al. | 2021 | 4 | 1 | 2 | good |
| Thomas et al. | 2022 | 4 | 1 | 2 | good |
| Otiti-Sengeri et al. | 2022 | 4 | 1 | 3 | good |
| Rosental et al. | 2021 | 4 | 1 | 2 | good |
| Kashif et al. | 2021 | 4 | 1 | 2 | good |
| Xu et al. | 2021 | 4 | 1 | 3 | good |
| Li et al. | 2021 | 4 | 1 | 3 | good |
| Yurttas et al. | 2021 | 4 | 1 | 3 | good |
